# Economic burden of PTSD in the UK: a systematic review and economic analysis

**DOI:** 10.1136/bmjopen-2024-084394

**Published:** 2025-07-22

**Authors:** Paul Montgomery-Marks, Siddhartha Bandyopadhyay, Clio Belle Weisman, Tamoghna Bose

**Affiliations:** 1 Department of Social Policy, Sociology and Criminology, University of Birmingham College of Social Sciences, Birmingham, UK; 2 Department of Economics, University of Birmingham College of Social Sciences, Birmingham, UK; 3 Social Work, Tulane University, New Orleans, Louisiana, USA

**Keywords:** HEALTH ECONOMICS, Anxiety disorders, Social Support, Stress, Psychological, Adult psychiatry, Psychosocial Intervention

## Abstract

**Abstract:**

**Objectives:**

Post-traumatic stress disorder (PTSD) constitutes a significant anxiety disorder that exerts substantial societal and familial impacts, while concurrently imposing an additional as well as a substantial burden on the healthcare system. Beyond the direct expenses incurred in its treatment, PTSD also gives rise to broader economic costs. The details of these costs in the UK are currently, we believe, unknown.

**Design:**

Our methodology was developed collaboratively with a collaborative advisory group of clinicians, patients, carers and other stakeholders. A comprehensive search strategy was devised to identify articles, including systematic reviews evaluating the economic costs linked to PTSD. We adhered to the National Institute for Health and Care Excellence checklist for economic evaluations. After applying our search strategy, the selected included papers were analysed to identify various cost categories contributing to the economic burden of PTSD.

**Data sources:**

PubMed, PsycInfo, PTSDpubs, EMBASE and Google Scholar were searched from January 1990 until January 2023; the search was revised and re-run in September 2024.

**Eligibility criteria for selecting studies:**

The articles must have been published originally in English and include a detailed evaluation of costs related to PTSD.

**Data extraction and synthesis:**

Two independent reviewers used standardised methods to search, screen and code included papers. After applying our search strategy, selected included papers were analysed to identify various cost categories contributing to the economic burden of PTSD. Detailed information on per-contact and per-session costs of healthcare variables was obtained at 2020/2021 prices. Additionally, with the advisory group, we ensured the validity of frequencies and unit cost figures associated with variables linked to PTSD. Further, indirect socio-economic costs arising from PTSD were computed.

**Results:**

By extrapolating from cost components identified, our findings indicate an average annual cost exceeding £14 780 per person. Given current 2020/2021 prevalence rates, this translates to an annual societal burden of £40 billion, a figure that does not encompass the many additional financial burdens stemming from PTSD, such as poor or inconsistent employment. This figure does not include the myriad intangible costs ranging from reduced quality of life to suicidality and countless other issues a person may suffer from as a result of PTSD. Finally, this number does not capture the breadth of impact, as it is difficult to quantify how the families, communities and social systems are adversely affected (both financially and otherwise) by the condition.

**Conclusion:**

The economic and societal burden of PTSD in the UK is far greater than what extant research and common understanding indicate, as there is minimal awareness and information relating to indirect costs or ancillary effects such as discrimination, joblessness, substance use and other comorbidities. Ultimately, we found that there exists, conservatively, an annual excess societal burden of £40 billion, or approximately £14 780 per person. We demonstrated that PTSD is a significantly larger burden on society and individuals than estimated and that we are gravely underquantifying the cost of this increasingly prevalent condition.

**STRENGTHS AND LIMITATIONS OF THIS STUDY**

- This research provides a reproducible, transparent and rigorous process for identifying and synthesising the available quantitative data on the economic burden associated with post-traumatic stress disortic Stress Disorder (PTSD).
- The study provides detailed cost categories encompassing the annualised societal burden associated with per-person PTSD.
- Certain costs are hard to measure, such as stigma and discrimination, for which we have provided only a qualitative analysis. These in turn may lead to indirect costs such as reduced income and higher dependence on social security assistance.
- Recognising the hidden costs of PTSD (eg, in criminal justice, family support services and education) for which we were unable to provide estimates suggests the actual economic burden is higher than estimated here.
- The study is limited to the UK, which limits generalisability to other countries

## Introduction

Post-traumatic stress disorder (PTSD) is an anxiety disorder that has received increased attention in recent years, in part due to a growing awareness of the burden it places at individual, familial and societal levels. The disorder may develop in a number of circumstances, most frequently as a result of witnessing or experiencing a direct traumatic event or undergoing a more systemic pattern of trauma or abuse. Such events may include physical violence, life-threatening injury, sexual abuse, military service which includes combat situations or repeated or extreme exposure to the details of traumatic events, such as the experiences of first responders, individuals tasked with collecting human remains or documenting the details of child abuse cases.^1^

Management of PTSD involves several psychological and pharmacological interventions, with an array of individual, agency and stakeholder opinions relating to these practices. The differing approaches are guided by evidence, clinical expertise, patient preferences and various clinical or treatment guidelines published by, for example, the US Department of Veterans Affairs (VA)^2^ and the National Institute for Health and Care Excellence (NICE),^3^ which are the two most comprehensive, used and research-supported guidelines in the UK and the US.^4^ These guidelines support and promote, first and foremost, individual trauma-focused psychotherapy which can be broken down into specific evidence-informed modalities.^25^,^7^ The American Psychological Association in their clinical practice guidelines recommends cognitive behavioural therapy (CBT), cognitive processing therapy (CPT) and prolonged exposure therapy (PET); their conditional recommendations are for brief eclectic psychotherapy, eye movement desensitisation and reprocessing (EMDR) and narrative exposure therapy. The medications suggested for treatment are selective serotonin reuptake inhibitors (SSRIs) such as fluoxetine, paroxetine, sertraline and, less frequently, serotonin and norepinephrine reuptake inhibitors like venlafaxine.

The management of PTSD imposes a substantial economic burden. In addition to the direct costs of PTSD treatment as described above, there are a number of associated costs. These relate to lack of employment, substance misuse among patients and loss of productivity at work. The impact on families and friends can be considerable, although often much harder to quantify. PTSD is frequently partially responsive, misdiagnosed, left untreated or unreported.^8^,^10^ Currently, wait times for diagnosis within the National Health Service (NHS) are around 2 years which increases the economic burden in itself, before treatment can even begin. The stigma surrounding mental health in general, and PTSD in particular, further contributes to these issues, and despite the guidelines, there is a lack of consistency and disparate levels of adherence.^11 12^ These factors only exacerbate the costs of PTSD as patients try one treatment after another.

The aim of this paper is to provide best estimates of the economic costs arising out of PTSD for patients in the UK. At the outset, we should be clear that estimating the costs poses several challenges. First, PTSD is often present with other mental health conditions, and disentangling the burden due to PTSD from other comorbidities is difficult. Second, there is considerable variation in the severity of PTSD, and studies that have captured different groups of patients present different unit costs. Third, as we mentioned above, PTSD is often left unreported and there are hidden costs in productivity loss and substance misuse. Fourth, the recent pandemic and its aftermath likely left patients, their families and frontline workers^13 14^ who have been heavily exposed to death and suffering with PTSD; it is impossible to know at this time the extent of new PTSD diagnoses stemming from COVID-19, as it takes time for the symptoms to manifest and present. Given overburdened healthcare systems, a considerable portion of such people is likely not being treated, thus creating a potentially large burden in the future. Finally, while there is prior research (eg, Scott *et al*^15^ and Foley and Massey)^16^ which has explored the prevalence of PTSD in the UK, these studies have not examined the financial or economic impact of PTSD in the UK. The healthcare system in the UK differs significantly from those in other countries, leaving a gap in the literature that we aim to fill with this paper. We have used our judgement on the cost components that we think must be included from studies in other countries and incorporated costs of such treatment in the UK. Where there is doubt, we have consulted widely with health and other professionals to ensure our estimates are in line with what practitioners confirm is realistic. Further, we have engaged with patients to ensure that the costs we have included have been relevant to them. With these caveats, we believe it will almost certainly underestimate the non-medical costs, but nonetheless we hope this provides a useful benchmark as the first study that calculates the per patient cost of PTSD in the UK.

To understand the economic burden, it is first necessary to know the prevalence of PTSD in the population. There is a far more detailed and extensive research base in the USA, thus we included it for comparison and context. According to a 2022 study by Jitender Sareen^17^ sourced from uptodate.com, the lifetime prevalence of PTSD in the adult population of the USA varies between 6.1% and 9.2%. Additionally, the 1-year prevalence rates are reported to range from 3.5% to 4.7%. In a military context, the Department of Defence surveys reveal positive screens for PTSD among about 17% of active Army soldiers and 25% of Army National Guard and Army Reserve personnel. Notably, those with multiple deployments tend to have higher rates.

In the UK, we did not find any study which computed the average prevalence of PTSD; however, we could ascertain a 7.4% prevalence in veterans as a whole, with higher rates existing in those soldiers who have been deployed versus those remaining in the UK.^18^ We used evidence of prevalence in the military and civilian population and computed the overall lifetime prevalence in the UK which stands at approximately 4% for 2020/2021. This is supported by websites such as PTSDUK.org (a charity focused on treatment and support) and the National Institutes of Health^19^ agree that there are an estimated 6 665 000 people expected to develop PTSD at some point in their life, and at any given time, approximately 4 in 100 people—or 2 612 000 individuals—are diagnosable with PTSD. Even more troubling is the suggestion that, primarily due to COVID, there will be a rise of 77 000 cases per year. Despite this, the disorder continues to be an incredibly misunderstood, often misdiagnosed and stigmatised condition. Evaluating the economic burden is crucial as it will inform policymakers about the importance of PTSD and the provision of care.

Unfortunately, the treatment guidelines laid down are not always, or even often, in line with the *actual* services received by those with a PTSD diagnosis, and there is a myriad of barriers, including costs, to effective and sustainable recovery from the debilitating symptoms experienced by individuals seeking treatment.^20^ Despite the guidelines laid down, there is a lack of consistency and disparate levels of adherence to treatment suggestions. The lion’s share of studies focusing on treatments for PTSD recommend further research, higher quality design and longer follow-up times. There is recognition within the field of significant limitations and gaps in the research. There is clearly a need for effective treatments, an example of which might include a novel treatment in the USA which provides a manualised therapy combined with methylenedioxymethamphetamine (MDMA). This approach has been rigorously tested^21^ and suggests that it may be highly efficacious with some remission of symptoms at 3 months and achieving complete remission at 1 year. While opponents to the treatment argue it is more expensive than alternatives, it could, in the long term, ease the overwhelming weight of PTSD, as symptom remission (and in some cases, complete withdrawal of the diagnosis) may result in economic and psychosocial improvements not currently seen. This is particularly important as the social burden in general exceeds the direct healthcare costs, a point we will return to in our discussion.

## Methods

This section initially describes the procedure followed for conducting a review of evaluations of the economic costs associated with PTSD. In our literature review, we employed a checklist to ensure that the studies align with the NICE guidelines for conducting an economic evaluation which in turn informs the methodology for this study.^3^ To maintain comparability, it was essential that studies originating outside the UK were conducted within healthcare systems that essentially resemble the present UK NHS framework (NICE guidelines, see online supplemental appendix A.1). We primarily targeted studies that emphasised core cost categories: direct costs and indirect costs. It was crucial that these studies possessed a time horizon long enough to capture all pertinent cost and outcome disparities, which was a minimum of 12 months. Additionally, we assessed whether the studies articulated health effects in terms of quality-adjusted life years. By adhering to the NICE checklist for economic evaluation (see online supplemental appendix A.1), we aimed to produce findings in line with the decision-making processes of their guideline development group (GDG). Additionally, with the assistance of an advisory group comprising service users, clinicians, carers, third-sector service providers and policymakers, we verified the validity of the frequencies and unit cost figures for various variables associated with PTSD assessing face and content validity. The search strategy involved looking for all quantitative studies of any design (including systematic reviews) that evaluated the economic costs associated with PTSD. The search was conducted using key databases and search terms including economic losses, societal costs, financial burden and productivity losses (see the search strategy in an online supplemental file). Studies that were not published in English, did not evaluate societal/financial/economic losses or productivity loss or costs or burden to society were excluded.

Titles and abstracts were searched. The search was for the period 1990–2023 and could be for any country and then narrowed using the inclusion and exclusion criteria below. It was conducted in January 2023 and updated in September 2024.

### Inclusion and exclusion criteria

Studies published in English outlining the healthcare costs and wider non-healthcare costs (such as social care) in detail were included in our research (cost component details are explained in the variable section below). Along with the above-mentioned criteria, we checked by assessing its relevance to understand economic losses to society by following the NICE checklist criteria 1.1–2.2 (see online supplemental table A.1). Refer to table 1 to see the included studies. Earlier editions of updated papers and unpublished papers were excluded.

**Table 1:** Characteristics of the included studies

Authors	Condition	Aim	Sample	Method used
Davis *et al*^6^	PTSD	To estimate the economic burden of PTSD in the US civilian and military populations from a societal perspective.	n=11 828 145	Prevalence based human capital approach
Warth *et al*^23^	PTSD	To systematically review costs-of-illness studies and economic evaluations of therapeutic treatment for PTSD, and to assess their quality.	N.A. (31 studies included)	Systematic literature search
Ferry *et al*^24^	PTSD	To estimate the economic costs of PTSD among the Northern Ireland (NI) adult population.	n=74 935	Prevalence based, bottom-up study
Bothe *et al*^26^	PTSD	To add evidence regarding the quantity of monetary losses due to PTSD and to compare these costs to those incurred by non-exposed average insurant.	PTSD (n=12 887) and the No-PTSD Control Group (n=51 548)	Medical claims data were analysed for individuals with incident diagnoses of PTSD Results were compared with non-exposed average insurant matched on age and gender
Mavranezouli *et al*^25^	PTSD	To compare costs and quality-adjusted life-years (QALYs) of 10 interventions	PTSD (10 interventions had variable sample size, with each intervention having more than 100 individuals for the PTSD group)	Decision-analytical model was constructed to compare costs and QALYs of 10 interventions for adults with PTSD to no intervention.

PTSD, post-traumatic stress disorder.

### Study selection and data extraction

**Figure 1:**
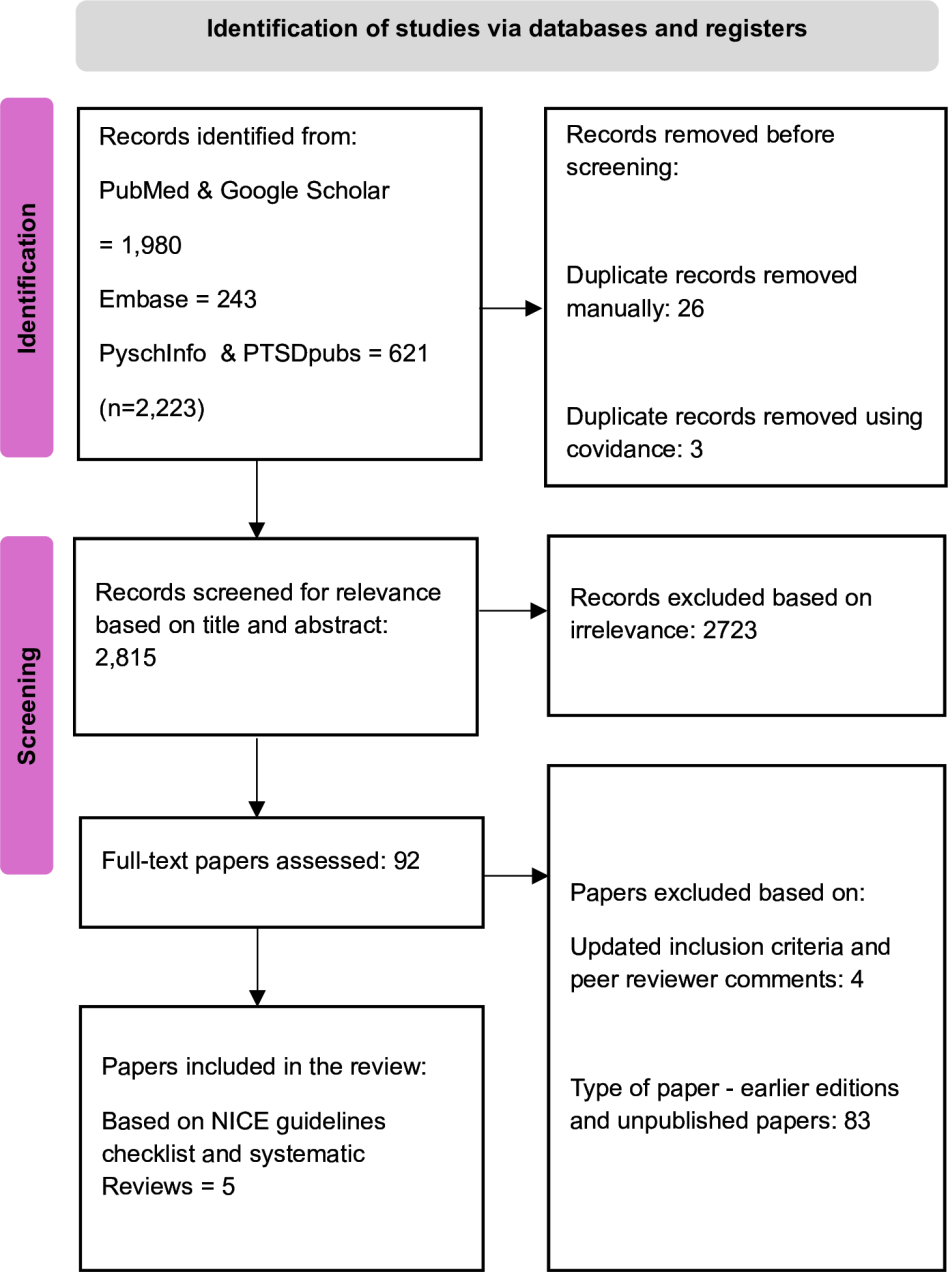
Selection of studies using the PRISMA flow chart. PRISMA, Preferred Reporting Items for Systematic Reviews and Meta-Analyses; NICE, National Institute for Health and Care Excellence.

Study selection was performed by one review author under the supervision of the other two authors for cross-check. Covidence software^22^ was used to store and code the retrieved records. Prior to the screening, all duplicate texts were removed. Titles and abstracts of the remaining texts were then screened for relevance. Full texts of the relevant papers were assessed for eligibility, and variables were extracted from all papers that met the inclusion criteria. Reference lists from published reviews were hand searched for relevant studies. See the Preferred Reporting Items for Systematic Reviews and Meta-Analyses flow chart below in figure 1.

Data on the following key variables were extracted from the included studies:

- Direct costs
  - Hospital costs (inpatient bed costs)
  - Medication costs
  - General practitioner (GP)/doctors
  - Treatment for alcohol abuse
  - Treatment for substance abuse
  - Psychiatrist
  - Psychologist
  - Counsellor
- Indirect costs
  - Homelessness cost
  - Disability living allowance
  - Unemployment allowance (ie, employment and support allowance)
  - Productivity loss at work, that is, absenteeism and presenteeism
  - Home-care worker
  - Social worker (mental health)
  - Premature mortality

The studies we included in our paper for understanding the cost analysis in the UK context are listed above in table 1, based on our selection procedure outlined in figure 1.

## Synthesis of the review

Warth *et al*,^23^ in their review of papers on the economic implications of PTSD, find most to be of low to moderate quality. The research by Davis *et al*^6^ has been especially useful and has provided a detailed breakdown of indirect costs as elucidated below. This paper, to our knowledge, is the first attempt to comprehend the economic costs of PTSD within the UK.

We derived costs from the included studies, which provided a comprehensive representation of the potential expenses for an individual living with PTSD, as different papers helped us come to grip with what components we should include in our cost decomposition. For instance, the paper by Davis *et al*^6^ was the sole publication that delved into the premature mortality associated with PTSD, including suicide; unfortunately, they combined all psychotherapy services which detracted from the specificity we are seeking. The paper by Ferry *et al*^24^ and Mavranezouli *et al*^25^ also facilitated our understanding of how frequently PTSD patients meet with various professionals critical to their treatment and recovery. The research by Ferry *et al*^24^ is particularly useful in their separating out visits to psychologists, psychiatrists and counsellors, all of whom fall under the umbrella of psychotherapeutic treatment providers, and Mavranezouli *et al*^25^ provided more recent cost estimates of such healthcare utilisation. Davis *et al*^6^ provided insights on the total excess costs of PTSD in 2018, estimated through a prevalence-based and human capital approach, drawing data from insurance claims, academic studies and government publications. Ferry *et al*^24^ aimed to determine the economic burden of PTSD in Northern Ireland’s adult population, using a prevalence-based, bottom-up approach, primarily using data from 74 935 participants in the Northern Ireland Study of Health and Stress. Bothe *et al*^26^ analysed claims data from a German research database to assess the direct and indirect costs associated with PTSD over a 5-year period. The study compared the costs for individuals with PTSD to those of non-exposed insurants of the same age and gender. Overall, the methodologies and findings of these studies highlight the substantial economic impact of health conditions and the necessity of accounting for both direct and indirect costs in health economic evaluations.

The economic burden of PTSD is substantial, but it is challenging to determine with precision due to varying calculation methods in the literature. Furthermore, the costs vary greatly for different population groups, and many of the hidden costs associated with undetected PTSD cannot be included in our estimates. We have arrived at the best possible estimates of healthcare costs through a synthesis of literature and expert opinion from stakeholders. However, these average costs mask considerable variation depending on the duration of diagnosis and treatment. Societal costs are even more difficult to estimate due to the presence of specific conditions (eg, unemployment and subsequent loss of tax revenue) in only a portion of the population. Therefore, we have weighted costs based on the estimated fraction of people with PTSD in the population who are experiencing the particular condition and prevalence rates, as discussed in the variable section below. Where possible, we have used CIs from the study, but otherwise we have done a sensitivity analysis by varying the costs by ±10%. Our review indicated a lack of specific studies on PTSD in the UK, and thus we have extrapolated from studies conducted in other countries while using appropriate unit costs for the UK. Prior studies in the UK have mainly focused on PTSD prevalence, overlooking the societal and economic burden or looking at specific groups, for example, nurses and police.

In our study, we obtained detailed information about the per-contact, per-session costs of various healthcare variables from the Personal Social Services Research Unit (PSSRU) website^27^ at 2020/2021 prices, the latest time point for which consistent data are available. We referred to the Ferry *et al*^24^ paper to estimate the annual frequencies of various contacts and visits for patients with PTSD. Prevalence rates for civilian^28^ and military^29^ populations were obtained from mental health counsellor consultations and digital newspaper reports. Further, for some variables, such as substance abuse disorder, unemployment allowance, home-care worker, loss of productivity at work and excess due to PTSD, we gathered data from Davis *et al*.^6^ To calculate the annual per-patient PTSD economic cost to the UK economy, we weighted the excess costs for PTSD compared with the general population with prevalence rates and population percentages.

While the study by Ferry *et al*,^24^ provided information on variables such as psychiatrists, psychologists and counsellors, we exercise caution when comparing and applying these findings to other populations and healthcare systems due to differences in estimation methodologies. Our study included the variable of premature mortality, as observed in the US study conducted by Davis *et al*.^6^

## Data analysis

Several studies have attempted to determine the economic burden of PTSD in various countries. In the USA, using data from 2018, the estimated annual cost of PTSD among civilians, active-duty military personnel and veterans was US$232.2 billion.^6^ Around 8% of the US population suffers from PTSD, with an annual cost of US$25 684 per patient for veterans, compared with US$18 640 for civilians. A study conducted in Germany found that the total costs due to PTSD were approximately €43 000 per individual, which is three times higher than the costs for non-exposed controls.^26^ A study by Ferry *et al*^24^ estimated that the total direct and indirect cost of PTSD in 2008 was £172 756 062, which is equivalent to £221 693 314.03 in 2020/2021 prices.^30^ With a sample size of 74 935, the estimated cost per patient with PTSD stands at around £3000 at 2020–2021 prices. This divergence in individual costs compared with our study can be attributed to several factors taken into account, including premature mortality, expenses related to substance abuse treatment, costs associated with homelessness and the inclusion of disability living allowances. The research conducted by Warth *et al*,^23^ which spanned across North America, Europe and Australia, incorporated 13 cost-of-illness studies and 18 economic evaluations. Their findings revealed a range in the annual direct excess costs from US$512 Purchasing Power Parity (PPP) to US$19 435 PPP. Additionally, the annual indirect excess costs were determined to be US$5021 PPP per person. The study also concluded that trauma-focused CBT, when combined with a selective serotonin reuptake inhibitor, proved to be more cost-effective compared with the standard treatment like talking therapies and medicine^31^ or no treatment at all. However, comparing these findings and generalising them to other populations and healthcare systems in different countries is limited due to the varying estimation methods used. We explain our variables and how we calculated our annual per-PTSD patient cost for each such variable in detail below:

### Direct costs

This looks at the direct costs of healthcare arising from PTSD.

#### Hospital costs

The annual expenses incurred by a PTSD patient in the UK for the year 2020/2021 have been assessed by estimating the cost of hospitalisation due to PTSD. This is calculated as the product of the number of hospital days a patient spends annually for PTSD-related health issues, the NHS per-bed day cost (£428) (sourced from the official PSSRU website^27^) and the percentage of PTSD patients (over and above the percentage of the non-PTSD population) requiring hospitalisation each year. The statistical figures for the proportion of PTSD patients hospitalised (3.2%) and the duration of their stays (31 days) are drawn from online supplemental file 4 of Mavranezouli *et al*.^25^ For the CI of the annual hospital costs, we considered a ±10% interval.

#### Medication costs

Paroxetine and sertraline are two examples of a variety of SSRIs recommended for the treatment of PTSD in adults and confirmed as in use among our consultation group provided by the NHS. Either one of the two medicines is prescribed to patients. The recommended dosage for both medications is one tablet per day for a minimum period of 12 months before gradually withdrawing it over a course of 4 weeks or longer,^31^ costing an average of £13.86 per patient. The cost for a PTSD patient’s consumption of paroxetine or sertraline in a year at 2018 prices is obtained from mental health medicine cost charts.^32^ Price inflator was used to convert to 2020/2021 prices.

#### GP/doctors

The PSSRU website provides details on the expense per consultation with an NHS GP during the 2020/2021 fiscal year, indicating a cost of £43.^27^ The frequency of GP visits for individuals with PTSD is extracted from S4 File of Mavranezouli *et al*.^25^ The number in the paper is derived by multiplying the average frequency of GP visits (nine times per year, with a range of 5–11) by the proportion of the PTSD population that accesses GP services over 12 months compared with the proportion of the non-PTSD population. These frequencies lined up with the experience of veterans and that of the GPs we consulted. Consequently, the excess societal cost of GP visits for each PTSD patient in a year can be estimated by multiplying the annual frequency of visits by the unit cost per consultation.

#### Treatment for alcohol abuse

Alcohol abuse is a significant issue for patients with PTSD,^33^ and a 50-minute consultation for preventing alcohol abuse costs £92.^27^ Information from various sources within the NHS indicates that the average recovery period from alcohol abuse is around 6–12 months.^34^ On advice from our consultation group, we obtained that, on average, a PTSD patient with alcohol abuse attends one meeting per week. Of the total UK population, 3.8%^28^ have served in the military at some point in their lives. As previously mentioned, the prevalence rate of PTSD in military personnel is 7.4%,^35^ while in the civilian population, it is approximately 4%.^35^ This rate was considered low by our consultation group, but given the low numbers in this group and the inevitable bias that such a group would add, we have decided to calculate based on these data from the NHS. According to the Davis *et al* study,^6^ 6.7% of civilian PTSD patients and 20.9% of military PTSD patients require alcohol abuse counselling. We calculated the treatment cost per PTSD patient for 2020/2021 by weighting the alcohol abuse percentages with the respective population and prevalence rates.

#### Treatment for substance abuse

Substance misuse and abuse frequently occurs in patients with PTSD.^6^ The cost per 50-minute consultation for substance abstinence programmes was reported to be £122^33^ for 2020/2021, as found on the PSSRU website. The frequency of sobriety meetings attended by PTSD patients is once per week, and treatment typically lasts a minimum of 6–12 months (information acquired through consultation with the mental health counsellor). By multiplying the excess percentage of civilian and military PTSD populations suffering from substance abuse, as obtained from the Davis *et al* paper,^6^ with the combined weights of population and prevalence, we calculated the annual adjusted cost of substance abuse abstinence programmes per patient for 2020/2021.

#### Psychiatry

The PSSRU website provided us with the consultation cost per hour of an NHS psychiatrist^27^ for 2020/2021 prices. In our paper, we converted the hourly cost to a 25-minute session cost, as this reflects the typical session length for a PTSD patient. On average, PTSD patients undergo 0.23 of these sessions per year.^24^ Ferry *et al* obtained this by calculating the frequency of psychiatric visits by PTSD patients over a 12-month period, dividing it by the sample size of PTSD patients in the study (74 935) and was more or less in line with the study by Mavranezouli *et al*.^25^ While these data are hard to visualise in common use, our consultation group thought that they were realistic. Therefore, the adjusted yearly cost of a PTSD patient, on average, is the product of the frequency of visits in a year and the per-session cost. For the CI of the annual costs related to visits to a psychiatrist, we considered a ±10% interval.

#### Psychology

The frequency of a PTSD patient’s visits to a psychologist over the course of a year is lower compared with those to a psychiatrist. Specifically, a PTSD patient visits a psychologist 0.14 times a year, which we obtained by dividing psychologist visits in a 12-month period by PTSD patients divided by the sample size of PTSD patients considered in the Ferry *et al* study,^24^ 74 935. The hourly rate of an NHS psychologist was obtained from the PSSRU website,^27^ which was later adjusted to the 25-minute session rate. The annual cost for a PTSD patient is calculated by multiplying the per-minute rate of the psychologist with the session length and the yearly frequency of visits. The patients and psychologists we talked to found it hard to assess the frequency of patient visits as they varied greatly across the country and over the course of the condition. We thus chose to base this calculation on Ferry *et al* and PSSRU data as it also accorded with Mavranezouli *et al*.^25^ In calculating the CI of the annual costs associated with a psychologist, we considered a ±10% interval.

#### Counsellors

CBT is regarded as an effective method for treating patients with PTSD, although success is limited as reported above.^4 6^ The hourly wage rate of a counsellor is recorded as £63 per hour.^27^ By applying the similar method by using statistics from Ferry *et al*,^24^ we have obtained that a PTSD patient visits the counsellors 0.5 times a year, and each session lasts for 50 min also consistent with figures in Mavranezouli *et al*.^25^ With this information, we can calculate the yearly cost to society for each PTSD patient’s counselling visits, similar to how we would for a psychiatrist or psychologist. For CI of the annual costs related to counselling, we considered a ±10% interval.

### Indirect costs

This computes the excess indirect cost, particularly the socioeconomic impact of PTSD.

#### Homelessness cost

Many PTSD patients face homelessness. In our paper, we obtained yearly shelter costs of £12 237 at 2020/2021 prices. On average, at any given time, 10% of PTSD patients experience homelessness. PTSD patients spend on average 46 weeks in shelters over their lifetime. This information was obtained from our consultation with our team of experts including a mental health counsellor. The adjusted homelessness cost due to PTSD is calculated, accounting for the weekly costs of shelter houses for 46 weeks and the percentage of the homeless PTSD population. For the CI of the annual costs related to homelessness, we considered a ±10% interval.

#### Disability living allowance

PTSD patients are entitled to receive disability living allowance. The UK government has set the weekly allowance for the year 2020/2021 at an average of £101.^36^ The average recovery period from PTSD is 9 months (information acquired through consultation with the mental health counsellor), with some patients recovering within 6 months, while others may take 12 months or more, according to information obtained from the internet. It has been found from the mental health counsellor consultation that 35% of PTSD patients are accessing the disability living allowance at any point in time. Therefore, to calculate the amount of disability living allowance accessed by a PTSD patient in a year at 2020/2021 prices, we multiply the weekly living allowance by the recovery period in weeks and by the excess percentage of PTSD patients accessing disability living allowances.

#### Unemployment allowance

In our analysis, we use Employment and Support Allowance Click or tap here to enter text as a proxy for measuring the economic burden of unemployment among PTSD patients. This benefit is available to PTSD patients during their treatment period. We obtained the per week benefit rate of £76.35^37^ from the UK government website. As previously noted, the average treatment duration for PTSD patients is on average 6–12 months. We derived the proportion of the PTSD population unable to remain employed, which is 13.1%, by combining data from a US 2022 paper^6^ and weighted population and prevalence figures. The adjusted economic cost of unemployment for an average PTSD patient for a year is calculated as the product of the weekly benefit rate, the average treatment duration (39 weeks) and the proportion of unemployed PTSD patients. This calculation is based on 2020/2021 prices.

#### Productivity loss at work

##### Absenteeism

The term absenteeism pertains to an employee’s regular non-attendance at their place of work. Based on the 2022 study^6^ on PTSD in the USA, it was found that an average PTSD patient loses 9.7 workdays per year due to PTSD-related absenteeism. The average hourly wage rate in the UK in 2020 is £15.15, with a typical workday consisting of 8 hours. Consequently, the loss in productivity due to PTSD-related absenteeism in a year is calculated as the product of the average hourly salary, the total number of work hours per day and the number of days of work missed. For the CI of the annual productivity loss due to absenteeism, we considered a ±10% interval.

##### Presenteeism

The term ‘presenteeism’ refers to the reduced productivity that occurs when employees attend work but do not perform at their optimal level due to poor health. According to the findings of Davis *et al*,^6^ an average PTSD patient results in a productivity loss (due to their condition) of 33.1 days per year for the economy. In a similar manner to the calculation for absenteeism, we determined the productivity loss per year per PTSD patient due to presenteeism. For the CI of the annual productivity loss related to presenteeism, we considered a ±10% interval.

### Home-care worker

The hourly rate for an NHS home-care worker, as reported on the PSSRU website,^27^ is £32. Home care involves aiding with daily living tasks and personal support to individuals or couples in their own homes. Davis *et al*^6^ reported that the average cost of caregiving per year for PTSD patients is 176 hours for civilian populations and 111 hours for military populations. The adjusted average for caregiving hours was found to be 172.1 hours per year. Again, our consulting group had wide variation in the amount of home care that was happening both between patients and across time. This being the case, we have decided that the most rigorous approach is to take the NHS data. The adjusted cost to society per year of home care for an average PTSD patient was calculated as the product of the adjusted annual average caregiving hours and the hourly rate for a home-care worker.

### Social worker

On average, a PTSD patient seeks assistance from a social worker 12 times per year, with each session lasting 45 min. The cost per hour for client-related activities is £90 (based on a 37.5 hour working week).^27^ The annual cost of social worker support per PTSD patient is calculated by multiplying the percentage of the PTSD population accessing these services (over and above the non-PTSD population) by the session length (in hours), the hourly rate (adjusted to 2020/2021 prices) and the annual frequency of visits. Data on session length, population percentage and annual visit frequency are sourced from Mavranezouli *et al*.^25^ To estimate the CI for annual costs due to social worker, a ±10% range was applied.^27^

### Premature mortality

We collected data from the Office of National Statistics for the age distribution of the UK population during the 2020/2021 period. We categorised age groups broadly as follows: 15–19, 20–24, 25–29, 30–34, 35–39, 40–44, 45–49, 50–54, 55–59, 60–64, 65–69 and 70–74. The reference age was set at 75 years. With an upper bound of 75, we calculated the potential years of life lost (PYLL) for each age group by subtracting the midpoint of the age range from 75. This PYLL value was then multiplied by the annual median salary to estimate potential earnings lost within each age bracket. In 2020/2021, the median annual salary in the UK was approximately £30 000, which we used to calculate the productivity loss for each year. Note that we discounted the PYLL value using an annual rate of 3.5% obtained from the NICE website. Average excess mortality was taken from the Davies *et al*^6^ paper and calculated separately for civilian and military populations and a weighted average of the two. Premature mortality was calculated for each age group by computing their excess age-specific mortality rate and summed using the fraction of the population in each age group multiplied by the discounted PYLL in that age group. We then multiplied by the average employment to population ratio and the median income of £30 000 as discussed above to get the average excess loss in productivity per person due to PTSD. This was not calculated separately per age group as the data for earnings did not match the intervals for which data were available for age-specific mortality rate. We also did a sensitivity analysis on this (see the sensitivity analysis section). The loss per person ranged from around £134 (using the upper bound of the excess death rate which was for the civilian population) to £534 (using the upper bound of the excess death which was for the military population), with the weighted average of the two giving us an estimated loss of £160 (refer to online supplemental tables A.2–A.5).

## Results

Table 2 presents a summary of the annual per PTSD patient societal cost calculated at 2020/2021 prices. The main findings of this study are summarised and explained as follows. The annual direct excess costs for a PTSD patient in the UK were estimated to be approximately £1118.41 (with a range of £840.91–£1353.02 per patient), encompassing various cost components such as hospital bed costs, medicinal costs, GP costs, treatment for alcohol and substance abuse and psychotherapy services like counsellors, psychiatrists and psychologists. The cost of bed usage, at slightly over £400 per visit, represents the largest proportion of direct annual costs. The cost of treatment for alcohol abuse is approximately £250, while treatment for substance abuse is around £190. However, when combined, the annual treatment cost typically amounts to £441.48, with a range of £294.32–£588.64. The costs associated with alcohol and substance abuse were estimated by factoring in the expenses related to attending sobriety meetings. The average frequency of GP consultations for a PTSD patient is calculated by the percentage of PTSD patients who visit a GP compared with the general population, conservatively assuming that those who go to the GP have the same frequency and multiplying by the cost of consultation (£43 per consultation). Administering medications like paroxetine or sertraline to PTSD patients incurs an annual cost between £16.43 and £11.29 per patient. The costs, in descending order, for psychotherapy services were £26.25 for counselling, £11.78 for psychiatry and £3.60 for psychology. For individuals with PTSD, counselling sessions occur once every 2 years, which is more than twice as frequent as visits to a psychiatrist and three times more frequent than consultations with a psychologist. For categories such as hospital costs, GP services and psychotherapy services, where only point estimates were provided and CIs were unavailable from our source references, we considered ±10% intervals for cost variations for robustness and reported the resulting costs in table 2.

**Table 2:** Different cost components associated with per-person PTSD at 2020/2021 price

Variables	Subcategories	Unit cost	Frequency	Excess cost
Direct costs	Hospital cost (inpatient bed costs)	£428/per bed-day (27)	Duration of stay (31 days) × percentage of PTSD population (3.2%) (±10%) (25)	£428 (£385.2–£470.8)
	Medication costs			
	Paroxetine 20 mg/sertraline 50 mg	£16.43-£11.29 /year (32)	1 medicine: 12 months	£13.86 (£16.43– £11.29)
	General practitioner/doctors	£43/consultation (27)	9 consultations (5–11 times) × PTSD population percentage (50%) (25)	£193.5 (£107.5–£236.5)
	Treatment for alcohol abuse	£92/contact (27)	50 mins per session: 9 months (6–12 months)—once a week Civilian: 6.7% Military: 20.9% 3.8% (50) military population (7.4% prevalence (44)) 96.2% civilian population (4% prevalence) Approximate adjusted percentage: 7%	£251.10 (£167.44–£334.88)
	Treatment for substance abuse	£122/contact (27)	50 mins per session - 9 months (6–12 months): once a week Civilian: 3.9% Military:10.7% Approx adjusted percentage: 4%	£190.32 (£126.88–£253.76)
	Psychiatrist	£2.05 /min (27)	25 mins session: 0.23 times (24) (±10%)	£11.78 (£10.60–£12.96)
	Psychologist	£1.03 /min (27)	25 mins session: 0.14 times (24) (±10%)	£3.60 (£3.24–£3.96)
	Counsellors	£1.05 /min (27)	50 mins session: 0.5 times (24) (±10%)	£26.25 (£23.62–£28.87)
Total direct costs				£1118.41 (£840.91–£1353.02)
Indirect costs	Homelessness cost		46 weeks (10%)	£1082.5 (£974.25–£1190.75)
	Disability living allowance	£101 /week (36)	9 months (6–12 months) (35%)	£1312.5 (£919.1–£1838.2)
	Unemployment Allowance (later Employment and Support Allowance	£74.35 /week (37)	9 months (6–12 months) Civilian: 13.5% Military: 7.85% Approx adjusted percentage: 13.1%	£376.95 (£251.3–£502.6)
	Productivity loss at work			
	Absenteeism	£15.15 /hour	9.7 days/year (±10%) (6)	£1175.64 (£1058.08–£1293.20)
	Presenteeism	£15.15 /hour	33.1 days/year (±10%) (6)	£4011.72 (£3610.54–£4412.89)
	Home-care worker	£32 /hour (27)	Civilian: 176 hrs/year (6) Military: 111 hrs/year Adjusted hours: 172.1 hrs/year	£5507.2 (£3552–£5632)
	Social worker	£90 /hour (27)	45 mins × 12 visits × PTSD population percentage (4.5%) (±10%) (25)	£36.45(£32.80–£40.10)
	Premature mortality	£160		£160 (£134–£534)
Total indirect costs				£13 662.96 (£10 532.07–£15 443.74)
Total costs				£14 781.37 (£11 372.98–£16 796.76)

PTSD, post-traumatic stress disorder.

The indirect costs of PTSD are classified into various categories such as homelessness costs, disability living allowance cost, unemployment cost, productivity loss at work costs, home care worker cost, social worker cost for mental healthcare and premature mortality cost. Large expenditures in this category include disability living allowance costs, averaging £1312.5 (with a range of £919.1–£1838.2), and homelessness costs at £1082.50. Homelessness affected approximately 10% of the PTSD population, a figure derived from the cost of shelter homes. Additionally, 35% of the PTSD population accessed disability living allowance benefits. We computed the unemployment cost by factoring in the employment and support allowance. Our analysis revealed that, on average, 13.1% of the PTSD population relies on unemployment benefits, resulting in an annual economic burden of approximately £376.95 per PTSD patient (with a range of £251.3–£502.6 per patient). The productivity loss due to absenteeism and presenteeism costs the economy £1100 and £4000 per patient, respectively. The cost of assistance from home care workers was found to be £5507.20 and is the largest contributor of indirect costs, while the cost of social workers catering to mental health needs, responsible for overseeing the needs of a PTSD patient, was the lowest contributor with £33.75 per year. The cost of premature mortality was computed based on the population distribution in England, which amounted to £160 (with the range of £134–£534 per patient). Similar to several categories in direct excess costs, in indirect excess costs, broader categories such as costs associated with homelessness, absenteeism, presenteeism, costs due to home-care workers and social care workers are considered with ±10% variations for robustness. Overall, the total indirect costs averaged £13 662.96 per patient, falling within a range of £10 532.07–£15 443.74 per patient.

**Figure 2:**
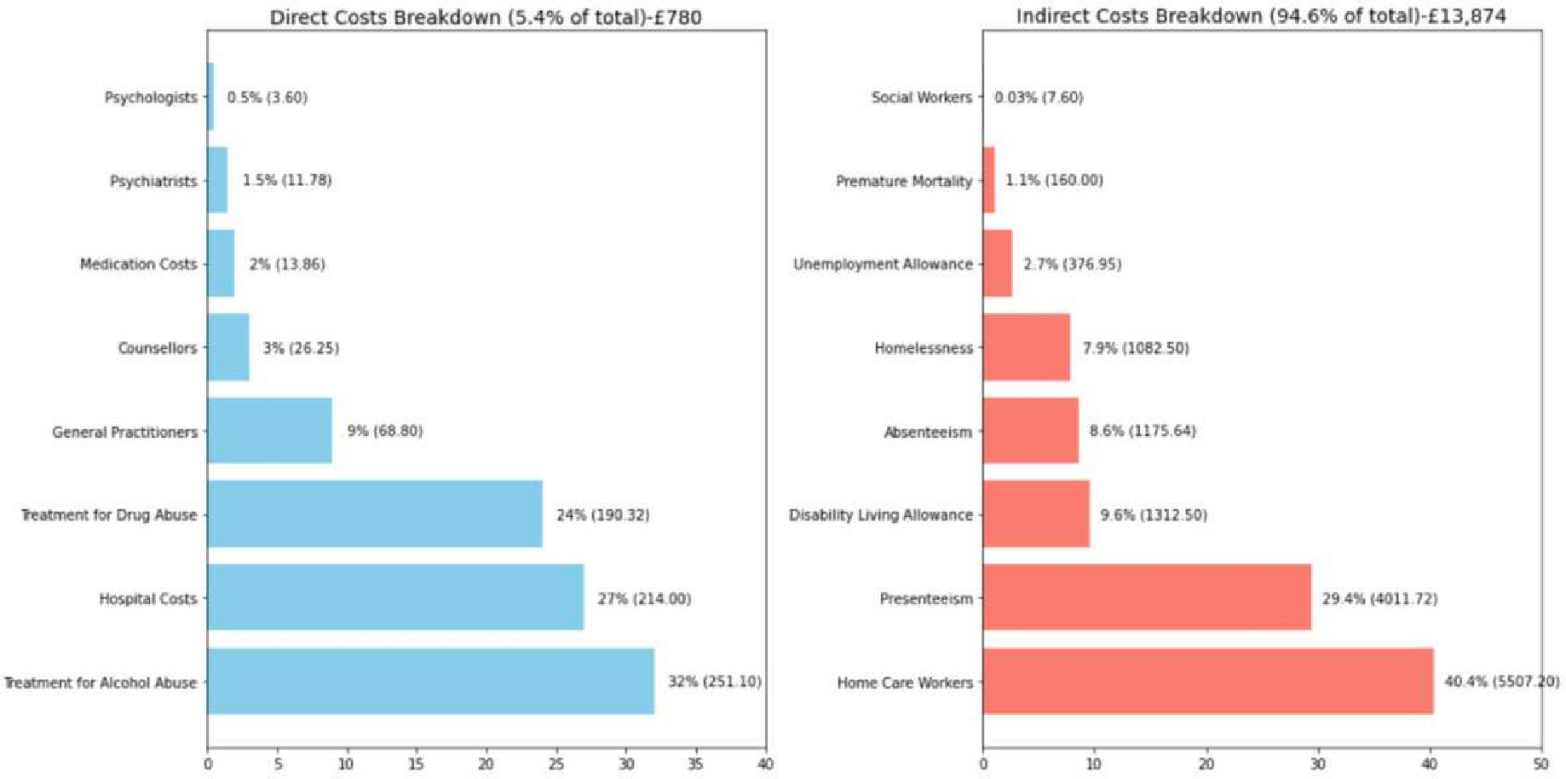
Various economic costs associated with post-traumatic stress disorder (note the actual costs are in parentheses and represented in £).

In summary, considering the major categories of direct costs and indirect costs, the estimated average annual cost per patient with PTSD in the UK is £14 781.37 with a range of approximately £11 372.98–£16 796.76. The diagrammatic representation of the various economic costs of PTSD is depicted below in figure 2. We used point estimates for the costs of certain variables, such as medication costs, treatment costs for substance abuse, disability living allowance costs and unemployment costs but with a ±10% variation. The economic burden analysis reveals significant cost components. Direct costs account for 7.6% of the annual per-person cost of PTSD, while indirect costs make up 92.4%. Among the direct costs, the largest contributors are hospital costs (38.2%), treatment for alcohol abuse (22.4%), GPs (17.3%) and treatment for drug abuse (17%). These are followed by counsellors (2.3%), medication costs (1.2%), psychiatrists (1%) and psychologists (0.6%). For indirect costs, the biggest portion comes from home care workers (40.3%), followed by presenteeism (29.3%), disability living allowance (9.6%), absenteeism (8.6%), homelessness (7.9%), unemployment allowance (2.75%), premature mortality (1.2%) and mental health social workers (0.35%).

### Sensitivity analysis

As explained in the discussion of results in table 2, a one-way sensitivity analysis was carried out to assess the robustness of the results by examining variations in category costs (eg, medicinal costs), treatment durations (ranging from 6–12 months) for issues such as alcohol or substance abuse, and allowances such as disability living allowance and unemployment benefits. We also considered unweighted and weighted excess premature mortality rates and weighted excess hours for home-care workers for patients suffering from PTSD. Furthermore, for categories concerning direct costs such as hospital expenses, GP services and psychotherapy services, as well as for indirect costs, including expenses related to homelessness, absenteeism, presenteeism and costs incurred from social care workers, interval estimates were unavailable. Therefore, we considered an interval of ±10% for robustness, as presented in table 2.

Following consultation with our team of experts and gathering information from NHS webpages, we used a treatment length of 6–12 months for recovery.^38^ This is consistent with a WHO report^39^ showing that up to 40% of people with PTSD recover within a year.

The figures for excess premature mortality rate were sourced from Davis *et al*,^6^ and we applied weightings based on PTSD prevalence rates within the UK’s civilian and military populations while analysing the impact on costs if these weightings changed due to changes in military and civilian prevalence rates. Further details on this methodology and the calculations can be found in the online supplemental appendix under Methodology for Computing Costs of Premature Mortality.

## Discussion

In this study, we estimated the economic burden of PTSD in the UK. The estimated cost is approximately £14 780. The major contributors to indirect costs of PTSD include home-care worker costs, loss of productivity at work, disability living allowances and homelessness. The major contributors to direct costs of PTSD include treatment for alcohol and drug abuse and hospital and GP visits. Additionally, there are other smaller yet significant factors that contribute to the economic burden of PTSD, such as psychotherapy service costs, which include psychiatrists, psychologists and counsellors, unemployment allowances, premature mortality, medication costs and social worker costs.

In our paper, we aim to discuss the need for more reliable estimates of PTSD costs. Linking PTSD cases to health and military records would help in this regard. PTSD and Complex Post Traumatic Stress Disorder (C-PTSD) are common in both serving and former military personnel. The rate of PTSD among UK veterans of all conflicts is estimated to be 7.4%, compared with 4% in the general population. Deployed veterans in Iraq and Afghanistan have higher rates of PTSD (9.4%) compared with non-participants in conflicts (5%). A study on UK armed forces found that 4% reported probable PTSD, while 19.7% reported other mental disorders, and 13% admitted to alcohol misuse.^35^ Some military personnel are reluctant to seek help due to the stigma associated with PTSD. In the words of the NHS: ‘the culture of the armed forces can make seeking help for a mental health problem appear difficult.’ Symptoms may appear years after leaving the military, and some veterans struggle silently. Suicides among military personnel in the UK are prevalent and may go unreported. Immediate support for managing trauma exposure is crucial, followed by comprehensive and accessible PTSD help for veterans. However, wait times for diagnosis and treatment, as noted earlier in this paper, significantly impact this issue.

To obtain more reliable estimates, one can also follow similar approaches used by Clarke *et al*^40^ in their study on Australian Military Personnel deployed in the Vietnam War. Their research revealed that most accepted disability claims did not occur during deployment or immediately after repatriation, but rather 20–30 years later. Eye and ear disorders, mental health conditions and musculoskeletal disorders were the most common causes of accepted disability claims. The duration of deployment was significantly associated with the prevalence of many disabilities. An important finding was the delayed nature of accepted disability claims among veterans, with a sharp increase observed after 1994, particularly for mental health and eye/ear conditions. It remains unclear whether these delayed effects are the result of war-related factors taking decades to emerge, age-related illnesses or changes in disability assessment policies in the ‘80s and ‘90s. Examples of policy changes include the recognition of PTSD as a disease as potentially war-related disabilities. Therefore, tracking disability claims can be another possible way in achieving reliable estimates about PTSD among veterans.

Martin *et al*^20^ provided treatment guidelines for PTSD, recommending different trauma-focused psychotherapies such as CBT, CPT, PET, image rehearsal therapy and EMDR. A study by Kar^41^ found CBT effective for acute and chronic PTSD across various traumas, comparable to other interventions. It is successfully applied in diverse populations, including children and online settings. However, despite its efficacy, non-response rates can reach 50%, (70% in veterans), influenced by factors like comorbidities. Evidence supports both psychological and neurophysiological bases for CBT response. While recognising its preventive potential, definitive recommendations for early CBT are inconclusive. The guidelines highlighted a lack of information regarding the targeted treatment of nightmares in PTSD, despite available psychological and pharmacological treatments. NICE guidelines indicate that EMDR is ineffective in combat trauma in marked contrast to all other included trauma types for which benefits were observed. However, the guidelines also note that there is very limited evidence to support the efficacy of EMDR.^3^

Treatment GDGs should consider producing more detailed recommendations for nightmare treatment due to its treatment-resistant nature and the increased risk of suicide. Our advisory group was clear that most patients had received multiple treatments for PTSD generally with limited effectiveness and talked of a ‘revolving door’ with patients returning over long periods for one intervention or another. The need for better treatment is manifestly required. Recently, a novel therapy combining MDMA combined with manualised therapy has shown promising results in the USA. The therapy has been rigorously tested^21^ and may offer enhanced efficacy, symptom remission and economic and psychosocial improvements. MDMA-assisted therapy demonstrates rapid onset of treatment efficacy, even in severe cases of PTSD and comorbidities such as dissociative PTSD, depression, substance use disorders and childhood trauma. It not only proves efficacious but also improves patient safety. Given the reported high success rate (68%) in the USA, this treatment has a demonstrable evidence base in terms of success rate and requires further evaluation.

## Limitations of our estimation

In addition to analysing the detailed costs associated with PTSD in this study, we acknowledge that there are certain costs related to PTSD that are difficult to quantify or measure. These represent some limitations in our cost estimates. We, however, provide a qualitative analysis of these hard-to-measure costs.

One significant cost is the stigma and discrimination faced by individuals with PTSD, which can result in limited opportunities and social exclusion. This, in turn, leads to indirect costs such as reduced income and increased reliance on social services. To illustrate this, a study conducted in Australia^42 42^ examined the economic impact of experiences of discrimination (EOD) on anxiety, depression, PTSD and psychological disorders. The findings indicated that these conditions accounted for 235 452 disability-adjusted life years (DALYs) lost in Australia due to EOD, with depression alone contributing to 31.3% of the total DALYs. In monetary terms, the estimated cost of EOD related to these health outcomes was approximately US$37.9 billion, equivalent to 3.02% of Australia’s average annual GDP (2001–2011). These findings align with similar studies conducted in the USA, emphasising the need for research on indirect costs associated with productivity loss and the potential benefits of reducing discrimination. In the UK, the military population accounts for 3.8% of the total population, and prevalence rates for the military and civilian populations are 7.4% and 4%, respectively. Consequently, when considering these adjusted rates, the overall prevalence rate in the UK is calculated to be approximately 4%. Using these rates, the estimated cost of EOD is US$21.8 billion in the UK.

Another concerning aspect is the high occurrence of PTSD among individuals involved in the criminal justice system. Several factors contribute to this, including being exposed to traumatic experiences and the stressful environment of incarceration. The expenses related to criminal justice involvement, such as law enforcement, court proceedings, imprisonment and probation, are significant. Moreover, individuals with PTSD may require specialised mental health services while in prison, further adding to the overall costs. When they are released, these individuals often encounter difficulties finding employment and reintegrating into society, leading to additional indirect expenses. A study conducted at an inner-city hospital in Atlanta, USA, discovered that nearly 31% of patients had PTSD, but only 13% had received prior treatment.^43^ The study also identified a correlation between trauma, PTSD and a higher likelihood of arrests, imprisonment and involvement in violent crimes. These findings indicate that PTSD-related behaviours are linked to the criminal justice system, emphasising the necessity for interventions and support to address these challenges.

PTSD can also have a significant impact on family relationships, leading to marital problems, family breakdowns and the need for child welfare services. These issues result in substantial social and economic costs. A study conducted in Alberta, Canada, focused on parents who experienced preterm birth (PTB) and the often overlooked non-financial costs associated with it.^44^ The study found that PTB is a traumatic event that disrupts ‘parents’ expectations of parenthood’, with the trauma stemming from factors such as prolonged uncertainty, lack of control, disruptions in belief systems and changes in expectations as parents. Activities like breastfeeding, kangaroo care and family-centred practices were found to help reconstruct parents' roles and provide a sense of control in the neonatal intensive care unit. The study suggests that healthcare providers should be educated about the symptoms of acute stress disorder and PTSD to better support parents, and future economic studies should consider the psychosocial implications of PTB to determine the total costs involved.

PTSD often causes issues educationally; it affects a person’s ability to concentrate, learn and perform academically, resulting in lower educational attainment. This, in turn, limits employment opportunities and earning potential, leading to substantial economic costs. A population-based cohort study conducted in Sweden by Vilaplana-Perez *et al*^45^ examined a large sample of 2 244 193 individuals. The study found that individuals diagnosed with PTSD were less likely to achieve educational milestones, including compulsory education and university completion, with the differences from the non-PTSD population being statistically significant. Importantly, this association held true even after considering factors such as shared familial characteristics among siblings, psychiatric comorbidity and general cognitive ability. These findings highlight the adverse impact of PTSD on educational outcomes and emphasise the need to address the barriers faced by individuals with PTSD in the education system. Compared with those without PTSD, individuals with PTSD had substantially lower odds of completing a certain level of education (87% lower), starting a university degree (68% lower) and finishing a university degree (73% lower). By providing appropriate support and resources, it may be possible to mitigate the long-term economic consequences of educational limitations associated with PTSD.

Recognising the hidden costs of PTSD is vital for formulating effective social policies and interventions. Addressing stigma and discrimination, improving support systems within the criminal justice system, strengthening family support services and promoting educational opportunities are all essential steps toward reducing the economic and social burden associated with PTSD. While our estimates do not account for these, this points out some of the further costs associated with PTSD, suggesting there are significant burdens over and above the costs we have computed.

## Conclusion

The economic burden of PTSD in the UK extends beyond direct healthcare and health-related expenses and encompasses substantial indirect expenses. Direct costs encompass various categories, including treatment costs for alcohol and substance abuse, expenses for accessing psychotherapy services such as counselling, psychiatry and psychology, medication costs and hospital and GP visits. Indirect costs encompass unemployment-related allowance, productivity losses at work, support from mental health social workers, homelessness cost, disability living allowance cost, home-care workers and the impact of premature mortality.

The paper also sheds light on additional costs that might contribute to the PTSD burden but are challenging to quantify. The UK would do well to consider the need for the collection of high-quality cost data to more accurately assess the different costs associated with this condition. These include the involvement of individuals affected by PTSD in criminal activities, the stigma and discrimination they face leading to social exclusion, the impact on personal relationships and the effect on education, potentially limiting future opportunities.

Furthermore, the paper explores various treatments for PTSD, highlighting a new and promising approach which seems to provide symptom remission. In conclusion, this paper underscores the need for increased awareness of PTSD, the development of more effective therapies and the expansion of evidence-based interventions to alleviate the substantial disease and economic burden of PTSD in the UK.

## Supplementary material

10.1136/bmjopen-2024-084394 online supplemental file 1

10.1136/bmjopen-2024-084394 online supplemental file 2

## Data Availability

Data are available upon reasonable request.
