# Supplementary material for: Economic burden of PTSD in the UK: a systematic review and economic analysis
Source: BMJ Open. 2025 Jul 22;15(7):e084394. doi: 10.1136/bmjopen-2024-084394 (PMC12306220; doi:10.1136/bmjopen-2024-084394)
Supplement: online supplemental file 1 [file bmjopen-15-7-s001.docx]

**APPENDIX**

NICE Checklist: Table [A.1](https://www.nice.org.uk/process/pmg6/resources/the-guidelines-manual-appendices-bi-2549703709/chapter/appendix-g-methodology-checklist-economic-evaluations#checklist-5)

| **Study identification**  *Include author, title, reference, year of publication* | | |
| --- | --- | --- |
| Guideline topic: | | Question no: |
| Checklist completed by: | | |
| **Section 1: Applicability (relevance to specific guideline review question(s) and the NICE reference case^[^**[**^a^**](https://www.nice.org.uk/process/pmg6/resources/the-guidelines-manual-appendices-bi-2549703709/chapter/appendix-g-methodology-checklist-economic-evaluations#ftn.footnote_1)**^]^)**  ***This checklist should be used first to filter out irrelevant studies.*** | **Yes/ Partly/ No /Unclear /NA** | **Comments** |
| 1.1 Is the study population appropriate for the guideline? |  |  |
| 1.2 Are the interventions and services appropriate for the guideline? |  |  |
| 1.3 Is the healthcare system in which the study was conducted sufficiently similar to the current UK NHS context? |  |  |
| 1.4 Are costs measured from the NHS and personal social services (PSS) perspective? |  |  |
| 1.5 Are non-direct health effects on individuals excluded? |  |  |
| 1.6 Are both costs and health effects discounted at an annual rate of 3.5%? |  |  |
| 1.7 Is the value of health effects expressed in terms of quality-adjusted life years (QALYs)? |  |  |
| 1.8 Are changes in health-related quality of life (HRQoL) reported directly from patients and/or carers? |  |  |
| 1.9 Is the valuation of changes in HRQoL (utilities) obtained from a representative sample of the general public? |  |  |
| 1.10 Overall judgement: Directly applicable/Partially applicable/Not applicable  There is no need to use section 2 of the checklist if the study is considered 'not applicable'. | | |
| Other comments:  .  .  .  . | | |
| **Section 2: Study limitations (the level of methodological quality)**  ***This checklist should be used once it has been decided that the study is sufficiently applicable to the context of the clinical guideline* ^[^**[**^b^**](https://www.nice.org.uk/process/pmg6/resources/the-guidelines-manual-appendices-bi-2549703709/chapter/appendix-g-methodology-checklist-economic-evaluations#ftn.footnote_2)**^]^.** | **Yes/ Partly /No/ Unclear/ NA** | **Comments** |
| 2.1 Does the model structure adequately reflect the nature of the health condition under evaluation? |  |  |
| 2.2 Is the time horizon sufficiently long to reflect all important differences in costs and outcomes? |  |  |

**Methodology for computing costs of premature mortality:**

As described in the main body, we collected data from the Office of National Statistics (ONS)for the age distribution of the UK population during the 2020/2021 period. Average economic loss from premature mortality was calculated by multiplying the age proportion of the population in England and multiplied by the (average) excess PTSD premature mortality rate, obtained from Davis et al. (2022). This was further multiplied by the average loss of potential earnings times the mortality rate for various age groups in England times the average employment rate (obtained from labour force survey) for 20/21. We categorised age groups broadly as follows: 15-19, 20 to 24, 25-29, 30 to 34, 35-39, 40 to 44, 45 to 49, 50 to 54, 55-59, 60 to 64, and 70-74. The reference age is considered as 75 years. By dividing the population within each group by the total UK population for that period, we determined the proportion for each age category. Similarly, ONS data provided us with age-specific mortality rates per 1,000 individuals in the UK for the year 2020. We normalised this rate per person. We used age-specific death rate per 1,000 population in the United Kingdom in 2020 with the population percentage to compute the weighted mortality rate across those age categories. We weighted the age specific mortality across gender by taking a weighted average (the percentage of females is 51% and the percentage of males is 49% in the UK population (taken from <https://www.ethnicity-facts-figures.service.gov.uk/uk-population-by-ethnicity/demographics/male-and-female-populations/latest/#:~:text=The%20data%20shows%20that%3A,up%2029.2%20million%20(49.0%25)>.) In 2020/2021, the median annual salary in the UK was approximately £30,000 which we used to calculate the productivity loss. With an upper bound of 75, we calculated the Potential Years of Life Lost (PYLL) for each age group by subtracting the midpoint of the age range from 75. This PYLL value was then multiplied by the annual median salary to estimate potential earnings lost within each age bracket. Note we discounted PYLL value using a rate of 3.5% obtained from the NICE website (<https://www.nice.org.uk/process/pmg20/chapter/incorporating-economic-evaluation>). Each table below looks at the costs from premature mortality under slightly different assumptions.

| Age group | Population Share | Mortality Rate | PYLL Value | Discounted PYLL | Median Earnings | excess mortality rate due to PTSD | Average Employment rate | Productivity loss due to premature mortality PTSD (undiscounted) | Productivity loss due to premature mortality PTSD (discounted) |
| --- | --- | --- | --- | --- | --- | --- | --- | --- | --- |
| 15-19 | 0.07513543 | 0.000198 | 58 | 25.54 | 30,000 | 0.6 | 0.55 | 8.54 | 3.76 |
| 20-24 | 0.08373563 | 0.000298 | 53 | 24.79 | 30,000 | 0.6 | 0.55 | 13.09 | 6.12 |
| 25-29 | 0.09094496 | 0.000447 | 48 | 23.89 | 30,000 | 0.6 | 0.55 | 19.32 | 9.61 |
| 30-34 | 0.09222682 | 0.000647 | 43 | 22.83 | 30,000 | 0.6 | 0.55 | 25.40 | 13.49 |
| 35-39 | 0.09014235 | 0.000996 | 38 | 21.57 | 30,000 | 0.6 | 0.55 | 33.78 | 19.17 |
| 40-44 | 0.08382681 | 0.001492 | 33 | 20.06 | 30,000 | 0.6 | 0.55 | 40.86 | 24.84 |
| 45-49 | 0.08774134 | 0.002339 | 28 | 18.28 | 30,000 | 0.6 | 0.55 | 56.89 | 37.14 |
| 50-54 | 0.09344936 | 0.003533 | 23 | 16.62 | 30,000 | 0.6 | 0.55 | 75.18 | 54.32 |
| 55-59 | 0.09071079 | 0.005225 | 18 | 13.65 | 30,000 | 0.6 | 0.55 | 84.46 | 64.05 |
| 60-64 | 0.07708725 | 0.008264 | 13 | 10.66 | 30,000 | 0.6 | 0.55 | 81.99 | 67.23 |
| 65-69 | 0.06714 | 0.01294 | 8 | 7.11 | 30,000 | 0.6 | 0.55 | 68.81 | 61.15 |
| 70-74 | 0.06785927 | 0.020414 | 3 | 2.89 | 30,000 | 0.6 | 0.55 | 41.14 | 39.63 |
| Total |  |  |  |  |  |  |  | 549.46 | 400.53 |

Table A.2: Productivity loss due to premature mortality PTSD by age groups taking a sample mean of civilian and military population (unweighted).

Note from the paper by Davis et al. 2022, we learned that PTSD-related premature mortality rates were 1.2 times for civilians and 1.8 times for the military compared to standard mortality rates. Calculating an unweighted average of these factors yielded a multiplier of 1.6 for PTSD-related excess mortality. Therefore, the excess mortality rate due to PTSD was calculated as 0.6. Using this excess mortality rate, combined with potential earning losses, the UK's average mortality rate, and population distribution across age groups, we calculated the premature mortality due to PTSD. The yearly per-person premature mortality from PTSD was then determined by summing these values across age groups. We found that the unweighted productivity loss due to premature mortality from PTSD, without discounting, is approximately £549 per person per year. When discounted, this loss reduces to around £400 per person per year.

In table A.3, we used the excess mortality rate due to PTSD for the civilian population (0.2) from Davis et al. 2022. We found that the weighted productivity loss due to premature mortality from PTSD for civilians, without discounting, is approximately £183 per person per year. When discounted, this loss reduces to around £134 per person per year per person.

| Age group | Population Share | Mortality Rate | PYLL Value | Discounted PYLL | Median Earnings | excess mortality rate due to PTSD | Average Employment rate | Productivity loss due to premature mortality PTSD (undiscounted) | Productivity loss due to premature mortality PTSD (discounted) |
| --- | --- | --- | --- | --- | --- | --- | --- | --- | --- |
| 15-19 | 0.07513543 | 0.000198 | 58 | 25.54 | 30,000 | 0.2 | 0.55 | 2.85 | 1.25 |
| 20-24 | 0.08373563 | 0.000298 | 53 | 24.79 | 30,000 | 0.2 | 0.55 | 4.36 | 2.04 |
| 25-29 | 0.09094496 | 0.000447 | 48 | 23.89 | 30,000 | 0.2 | 0.55 | 6.44 | 3.20 |
| 30-34 | 0.09222682 | 0.000647 | 43 | 22.83 | 30,000 | 0.2 | 0.55 | 8.47 | 4.50 |
| 35-39 | 0.09014235 | 0.000996 | 38 | 21.57 | 30,000 | 0.2 | 0.55 | 11.26 | 6.39 |
| 40-44 | 0.08382681 | 0.001492 | 33 | 20.06 | 30,000 | 0.2 | 0.55 | 13.62 | 8.28 |
| 45-49 | 0.08774134 | 0.002339 | 28 | 18.28 | 30,000 | 0.2 | 0.55 | 18.96 | 12.38 |
| 50-54 | 0.09344936 | 0.003533 | 23 | 16.62 | 30,000 | 0.2 | 0.55 | 25.06 | 18.11 |
| 55-59 | 0.09071079 | 0.005225 | 18 | 13.65 | 30,000 | 0.2 | 0.55 | 28.15 | 21.35 |
| 60-64 | 0.07708725 | 0.008264 | 13 | 10.66 | 30,000 | 0.2 | 0.55 | 27.33 | 22.41 |
| 65-69 | 0.06714 | 0.01294 | 8 | 7.11 | 30,000 | 0.2 | 0.55 | 22.94 | 20.38 |
| 70-74 | 0.06785927 | 0.020414 | 3 | 2.89 | 30,000 | 0.2 | 0.55 | 13.71 | 13.21 |
| Total |  |  |  |  |  |  |  | 183.15 | 133.51 |

Table A.3: Productivity loss due to premature mortality PTSD by age groups for Civilians.

Now similar to table A.3, in table A.4 we used the excess mortality rate due to PTSD for the military population (0.8) from Davis et al. 2022. We found that the weighted productivity loss due to premature mortality from PTSD for military, without discounting, is approximately £732 per person per year. When discounted, this loss reduces to around £534 per person per year, varying across age groups.

| Age group | Population Share | Mortality Rate | PYLL Value | Discounted PYLL | Median Earnings | excess mortality rate due to PTSD | Average Employment rate | Productivity loss due to premature mortality PTSD (undiscounted) | Productivity loss due to premature mortality PTSD (discounted) |
| --- | --- | --- | --- | --- | --- | --- | --- | --- | --- |
| 15-19 | 0.07513543 | 0.000198 | 58 | 25.54 | 30,000 | 0.8 | 0.55 | 11.39 | 5.02 |
| 20-24 | 0.08373563 | 0.000298 | 53 | 24.79 | 30,000 | 0.8 | 0.55 | 17.46 | 8.17 |
| 25-29 | 0.09094496 | 0.000447 | 48 | 23.89 | 30,000 | 0.8 | 0.55 | 25.76 | 12.82 |
| 30-34 | 0.09222682 | 0.000647 | 43 | 22.83 | 30,000 | 0.8 | 0.55 | 33.87 | 17.98 |
| 35-39 | 0.09014235 | 0.000996 | 38 | 21.57 | 30,000 | 0.8 | 0.55 | 45.03 | 25.56 |
| 40-44 | 0.08382681 | 0.001492 | 33 | 20.06 | 30,000 | 0.8 | 0.55 | 54.48 | 33.12 |
| 45-49 | 0.08774134 | 0.002339 | 28 | 18.28 | 30,000 | 0.8 | 0.55 | 75.85 | 49.52 |
| 50-54 | 0.09344936 | 0.003533 | 23 | 16.62 | 30,000 | 0.8 | 0.55 | 100.24 | 72.43 |
| 55-59 | 0.09071079 | 0.005225 | 18 | 13.65 | 30,000 | 0.8 | 0.55 | 112.61 | 85.40 |
| 60-64 | 0.07708725 | 0.008264 | 13 | 10.66 | 30,000 | 0.8 | 0.55 | 109.32 | 89.64 |
| 65-69 | 0.06714 | 0.01294 | 8 | 7.11 | 30,000 | 0.8 | 0.55 | 91.74 | 81.54 |
| 70-74 | 0.06785927 | 0.020414 | 3 | 2.89 | 30,000 | 0.8 | 0.55 | 54.86 | 52.85 |
| Total |  |  |  |  |  |  |  | 732.61 | 534.04 |

Table A.4: Productivity loss due to premature mortality PTSD by age groups for Military.

In table A.5, we calculated the Weighted - excess mortality rate due to PTSD, which is calculated as (military-excess rate mortality rate due to PTSD*(share of population who are military *military prevalence rate / ((share of population who are military *military prevalence rate)+ (share of population who are civilian *civilian prevalence rate))+civilian-excess rate mortality rate due to PTSD* (share of population who are civilian *civilian prevalence rate/((share of population who are military *military prevalence rate)+ (share of population who are civilian *civilian prevalence rate))). The civilian population percentage is 96.20% with the PTSD prevalence rate of 4% and the military population percentage is 3.80% with the PTSD prevalence rate of 7.40%. The excess mortality rate due to PTSD for civilian and military is 0.2 and 0.8 respectively. Using the formula above the weighted excess mortality rate due to PTSD is approximately 0.24. We found that the weighted productivity loss due to premature mortality from PTSD, without discounting, is approximately £219 per person per year. When discounted, this loss reduces to around £160 per person per year, varying across age groups.

| Age group | Population Share | Mortality Rate | PYLL Value | Discounted PYLL | Median Earnings | excess mortality rate due to PTSD | Average Employment rate | Productivity loss due to premature mortality PTSD (undiscounted) | Productivity loss due to premature mortality PTSD (discounted) |
| --- | --- | --- | --- | --- | --- | --- | --- | --- | --- |
| 15-19 | 0.07513543 | 0.000198 | 58 | 25.54 | 30,000 | 0.24 | 0.55 | 3.42 | 1.50 |
| 20-24 | 0.08373563 | 0.000298 | 53 | 24.79 | 30,000 | 0.24 | 0.55 | 5.24 | 2.45 |
| 25-29 | 0.09094496 | 0.000447 | 48 | 23.89 | 30,000 | 0.24 | 0.55 | 7.73 | 3.85 |
| 30-34 | 0.09222682 | 0.000647 | 43 | 22.83 | 30,000 | 0.24 | 0.55 | 10.16 | 5.39 |
| 35-39 | 0.09014235 | 0.000996 | 38 | 21.57 | 30,000 | 0.24 | 0.55 | 13.51 | 7.67 |
| 40-44 | 0.08382681 | 0.001492 | 33 | 20.06 | 30,000 | 0.24 | 0.55 | 16.34 | 9.94 |
| 45-49 | 0.08774134 | 0.002339 | 28 | 18.28 | 30,000 | 0.24 | 0.55 | 22.76 | 14.86 |
| 50-54 | 0.09344936 | 0.003533 | 23 | 16.62 | 30,000 | 0.24 | 0.55 | 30.07 | 21.73 |
| 55-59 | 0.09071079 | 0.005225 | 18 | 13.65 | 30,000 | 0.24 | 0.55 | 33.78 | 25.62 |
| 60-64 | 0.07708725 | 0.008264 | 13 | 10.66 | 30,000 | 0.24 | 0.55 | 32.80 | 26.90 |
| 65-69 | 0.06714 | 0.01294 | 8 | 7.11 | 30,000 | 0.24 | 0.55 | 27.52 | 24.46 |
| 70-74 | 0.06785927 | 0.020414 | 3 | 2.89 | 30,000 | 0.24 | 0.55 | 16.46 | 15.85 |
| Total |  |  |  |  |  |  |  | 219.78 | 160.21 |

Table A.5: Productivity loss (weighted by Civilian and Military population) due to premature mortality PTSD by age group
