# Supplementary material for: Economic burden of PTSD in the UK: a systematic review and economic analysis
Source: BMJ Open. 2025 Jul 22;15(7):e084394. doi: 10.1136/bmjopen-2024-084394 (PMC12306220; doi:10.1136/bmjopen-2024-084394)
Supplement: online supplemental file 2 [file bmjopen-15-7-s002.docx]

**Search Strategy:**

Searches of the following databases were conducted: PubMed, Embase, PTSDpubs PsycInfo and Google Scholar.

Below are the search terms utilised for these databases. They were generated by consulting with experts and by reference to all previous reviews in the area. We did not deviate from our search strategy or terms, maintaining continuity for the duration of the project.

Titles and abstracts were searched. The search was for the period 1990-2023 without exclusion by country. It was first conducted in January 2023 and updated in September of 2024.

Search Terms:

- 1. ("economic loss*" OR "economic burden*" OR "economic cost*" OR "societal cost*" OR "financial burden*" OR "financial loss*" OR "financial cost*" OR "productivity loss*" OR "indirect cost*" OR "monetary cost*" OR "economic impact")
  2. ("post-traumatic stress disorder" OR "posttraumatic stress disorder" OR PTSD OR "combat stress" OR "traumatic stress" OR "psychological trauma" OR "post-traumatic stress" OR "acute stress disorder" OR "complex PTSD" OR "PTSD symptoms")
  3. AND/1-2

Inclusion and exclusion criteria also remained the same for the search conducted in September of 2024. These criteria are stated below but are summarised in the Methods section of the paper.

Inclusion and exclusion criteria:

Studies published in English outlining the health and social care costs and other non health care costs in detail were included in our research (cost component details are explained in the variable section of the paper). Along with the above-mentioned criteria, we checked by assessing relevance to understand economic losses to society by following the NICE checklist criteria 1.1 to 2.2 (see table A.1). Refer to table 1, to see the included studies. Earlier editions of updated papers and unpublished papers were excluded.

Study selection and data extraction:

Study selection was performed by one review author under the supervision of the other two authors for cross-checking. The Covidence software (22) was used to store and code the retrieved records. Prior to the screening all duplicate texts were removed. Titles and abstracts of the remaining texts were then screened for relevance. Full texts of the relevant papers were assessed for eligibility, and variables were extracted from all papers that met the inclusion criteria. Reference lists from published reviews were hand searched for relevant studies. See the PRISMA flow chart (figure 1).
