# Supplementary material for: Economic burden of PTSD in the UK: a systematic review and economic analysis
Source: BMJ Open. 2025 Jul 22;15(7):e084394. doi: 10.1136/bmjopen-2024-084394 (PMC12306220; doi:10.1136/bmjopen-2024-084394)
Supplement: Supplementary file 3 [file bmjopen-2024-084394.reviewer_comments.pdf]

PEER REVIEW HISTORY

BMJ Open publishes all reviews undertaken for accepted manuscripts. Reviewers are asked to complete a checklist review form and are provided with free text boxes to elaborate on their assessment. These free text comments are reproduced below.

ARTICLE DETAILS

Title (Provisional)

The Economic Burden of PTSD in the UK: A Systematic Review and Economic Analysis

Authors

Montgomery, Paul; Bandyopadhyay, Siddhartha; Weisman, Clio Belle; Bose, Tamoghna

---

VERSION 1 - REVIEW

**Reviewer**	**1**
**Name**	**McGowan, Iain**
**Affiliation**	**QUB, School of Nursing & Midwifery**
**Date**	**27-Mar-2024**
**COI**	**None**

Thank you of asking me to review this paper, and also for accommodating my other commitments to allow extra time for the review. The authors seek to calculate the societal cost of PTSD in the United Kingdom using the 20/21 financial year as a reference year. In order to do so they identify the prevalence of PTSD the UK, as well as identify salient cost categories through a literature search. Extrapolation from the included literature provides an estimate of a societal cost of circa £40000/ per person with PTSD in the UK. This they estimate extrapolates to a total societal burden in excess of £100 billion which they acknowledge is likely to be an underestimate. In general I think the authors have attempted to address a very pressing issue and have made a good attempt at doing so. There are however several issues with the paper that I think should be addressed prior to publication.

I found this paper, at times, a difficult paper to read. I think the paper is too long (circa 10,000 words) and there is a degree of over complexity in the methods used and it can be difficult to follow the authors decisions and processes used in the paper. There are also some inconsistencies in the paper that if addressed should enhance the paper.

The introductory section attempts to set the scene in terms of the international literature around PTSD and is very detailed and becomes confused, particulalry around page 3 para 2 where the authors start to detail how they calculated the UK prevalence of PTSD. I would

have expected this in the methods section and not in an introductory section. In this vein, the methods used to calculate the prevalence rate seem very complex. The authors draw the readers attention to the papers by Scott et al (2022), Foley & Massey (2020) and Yunitiri et al (2021) that report prevalence of PTSD in the UK. Given the three peer reviewed papers in the last four years reporting prevalence of PTSD in the UK it is not clear why the authors chose to compute their own prevalence rate. A formula is provided but it is not clear which figures are used to calculate the final prevalence rate. I am also unclear on the relevance of the international literature reported in the context of detailing what was done to estimate prevalence of PTSD in the UK.

Also, in relation to the introduction I am not sure what relevance the discussions on treatments for PTSD adds to the paper. This is a personal issue and doesn't detract from the overall quality of the paper, but acknowledging my contention that the paper is too long, the authors may want to reconsider how they present this information. Related, the authors correctly assert that there are four medications suggested for treatment by the APA, however to the best of my knowledge only paroxetine and sertraline are licensed in the UK for treating PTSD. I think it would be appropriate to draw readers attention to that.

In the methods section, there are some issues with consistency and I think the purpose of the review of the valuations is lost in the narrative. Page five suggests that the review is to be used to identify categories to decompose the costs associated with PTSD. I assume this means that they will look at what others have costed and use this as a guide to identity cost categories for they analysis. It is not clear, firstly, if this search is conducted as a systematic literature review. If so, I would suggest that the review is incomplete. Arguably at a minimum the PTSD Pubs database should be included in any search strategy-regardless of the presentation or otherwise as a systematic review. I would also suggest that at least one database with a psychological focus (PsychInfo or PsychLit) should be part of the search strategy. I would also question the relevance of EMBASE given the nature of the paper and pharmaceutical focus of EMBASE.

Secondly, the search strategy "involved looking for systematic reviews...." (p5). This is not expanded upon in the inclusion criteria section. If it is the case that only systematic reviews are to be included then it would suggest that none of the papers in the review meet the inclusion criteria. Again, on consistency, the narrative on page 5 suggests the terms 'productivity losses' is one of the search terms but this does not appear in the search strategy. Likewise, I would have expected other PTSD associated MESH terms to be included to ensure a fuller representation of the current evidence base.

Thirdly, I understand why the authors have sought to broaden the evidence in relation to cost categories given the relative paucity of evidence in relation to PTSD, however I need more convincing that the health conditions chosen are the most relevant given that PTSD is associated with a myriad of other mental and physical health conditions including schizophrenia and some cancers as well as those highlighted by the authors. It would also be

helpful if an additional column could be added to table 1 detailing the cost categories identified from the included studies. It is not clear from the paper the additional benefit of exploring these non PTSD health conditions over simply looking at the three included papers focussing on PTSD. An additional column would help in this regard.

The confusion around the aim of the review is compounded in the synthesis section. The justification for looking at other health conditions is that a systematic review of PTSD economic papers (Warth et al, 2020) reported that their included studies were of low to moderate quality. I am not sure that this is a justified criticism given that two of the three PTSD studies the authors include were not reported in the der Warth paper. It is noteworthy that the authors make no comment on the quality of the other health condition papers in their review.

There are also sections dealing with methodology reported in the synthesis section. Unfortunately, I think this section needs completely rewritten and, ideally, simplified. For me, it would be simpler for the reader (and probably the authors!) to use the cost categories reported in the three PTSD papers found in their review without reference to the other health conditions unless there is a major reason why they have to be included. The authors may want also to consider restructuring the methods section way from the systematic review format, rather just report what they did without the constraints of a formal SR.

The section on the calculation of economic costs of PTSD is good-notwithstanding my concerns above-but again overly complex in its presentation. For example, in the data analysis on page 10 I would expect the two paragraphs directly above the sub heading to be in there, not a discussion on previous literature, which sits better in an introductory section. That said, the level of detail in the cost categories is good and the detail provided is what is needed for the reader to follow the decision making of the authors.

Some minor points in relation to cost categories; the cost of psychiatric care and psychology, I think, should be categorised as direct healthcare cost. I am not sure my psychiatrist colleagues would take kindly to being categorised as non health care giving, given that GPs and other Doctors have been classified as health care providers and that they are also GMC regulated. I could be persuaded around counselling but as the brunt of the treatments listed are borne by the NHS they should be reflected as directed healthcare cost. I would argue treatment for drug and alcohol use would also fit into this category.

It appears that the authors appear to have assumed that both Paroxetine and Sertraline will be given to all clients with PTSD. This is highly unlikely for two reasons. Firstly, it would be very unusual for two SSRI's to be prescribed concurrently, so clients would get one or other but not both. Secondly, NICE guidelines indicate that medications should not be offered as a first line treatment for PTSD. Economic modelling also showed SSRIs are less cost effective than EMDR, brief individual trauma-focused CBT or self-help with support (NICE 2018). This doesn't make much difference to the overall results but to maintain some form of clinical credibility some comment needs to be made.

I enjoyed reading this paper, but it was hard work! I would strongly encourage the authors to consider simplifying the paper. These suggestions are made with the intention of improving the paper. I trust they will be received as such.

**Reviewer**	**2**
**Name**	**Marseille, Elliot**
**Affiliation**	**Health Strategies International**
**Date**	**22-May-2024**
**COI**	**None.**

Summary of article: This study aimed to estimate the economic burden of post-traumatic stress disorder (PTSD) in the United Kingdom. The authors conducted a comprehensive literature review to identify cost components associated with PTSD, including direct healthcare costs, direct non-healthcare costs, and indirect costs. They then calculated the annual per-patient cost for each component using UK-specific data and prevalence rates where available and adjusting data from other countries when necessary. The study reveals that the average annual cost per PTSD patient in the UK exceeds £40,000, with a total annual societal burden of approximately £100 billion.

The use of non-UK data is a common approach in modeling studies when country-specific data are not available. In this case, the authors have taken steps to mitigate the potential impact of using non-UK data by applying UK-specific unit costs and consulting with stakeholders to verify the validity of frequencies and unit cost figures. While there may be some differences in the frequencies and prevalence rates between the UK and other countries, the overall approach is reasonable given the data limitations.

Minor comments

Throughout, please make it clear when you are referring to costs of patients with PTSD and when you are referring to the excess costs of patients with PTSD, i.e. the costs attributable to PTSD.

Bottom 5 / top 6. Define direct health care costs direct non-health care costs and indirect costs.

Page 10, para 2: Word missing after “excess”?

Table 1. Why isn’t Warth 2020 included?

Page . 12. I would have thought that psychiatrists, psychologists and councilors administering cognitive behavioral therapy would all be included in direct medical care costs rather than as a non-health care cost**.** I am more familiar with the healthcare system in the U.S. where therapy of this type would be included as a direct cost of care. Perhaps conventions are different in the UK; otherwise, please move this to the direct health care cost section.

The manuscript would benefit from a thorough proofreading to address minor grammatical and typographical errors.

Major comments

Page. 6. The rationale for evaluating the economic costs associated with a wide variety of other (non-PTSD) mental health and non-mental health conditions is unclear. The stated rationale is to alert the authors to categories of costs that might be overlooked in the specific PTSD studies. But if they are not quantified in the PTSD studies how does this help? Providing general “context” for the actual PTSD analysis is an insufficient rationale. Were any papers other than the PTSD papers listed in table one actually used to make the final estimates? Another way of asking this question is, “Why include anything other than PTSD in #2 of the search strategy”?

Bottom of page 8 through line 50 of p 9: Further to my comments above, It is unclear how this review of the economic impact of non-PTSD conditions contributes to the current analysis. I recommend deleting it from what is already a long paper.

Page 11. Hospitalization costs. The explanation of these methods is not entirely clear to me. The text refers to the number of hospital visits due to “PTSD-related mental health issues”. However we know that PTSD, apart from mental health disorders, is implicated in a wide range of chronic diseases.

Page 11. Medication costs and GP costs. Similar to the point made above about hospitalization costs, why restrict medication costs to two SSRIs since again, PTSD is implicated in a wide range of conditions that require medications? Similarly, why restrict the analysis of doctor visits to GP care and not specialist care as well?

The general point is, why not simply compare the overall direct healthcare costs of people with PTSD to those without PTSD? It would appear that the current methods would systematically underestimate the true excess costs of direct health care for patients with PTSD. Indeed, the £312 annual cost of direct excess healthcare for a PTSD patient in the UK just *seems* very low. It represents less than a 10% increase over average per capita healthcare spending in the UK in 2022.

Page 15. Calculation of premature mortality. According to the explanation given it appears that the median annual salary of £30,000 was applied for the full life expectancy of 75 years. However, the average retirement age is considerably below 75. Please explain or correct this calculation. Presumably these costs are discounted, but discounting is not mentioned.

Table 2 Why no entry of premature mortality cost estimates in the total cost column of Table 2?

A significant limitation is the lack of **sensitivity analyses** to assess the impact of varying key parameters on the cost estimates. Given the uncertainty surrounding some of the input data, it would be informative to present a range of cost estimates based on different assumptions or scenarios. For example, the single biggest contributor to PTSD costs is the economic losses associated with premature mortality. The authors rely on Davies 2022 which in turn seems to cite Miloyan 2016 for its estimate of excess risk of death of 1.2. However, there are studies such as Ahmadi 2011 that provide higher estimates of the relative mortality risk. Since

premature mortality is such a large driver of results, this is likely to be a difference that makes a difference. Providing this and other sensitivity analyses using published confidence intervals from the source articles where possible, would provide a more comprehensive understanding of the potential economic burden of PTSD in the UK. The provision of only the base case estimate of £40,000 is insufficient.

Overall assessment

Despite these limitations, the study makes a valuable contribution to the literature by providing a detailed breakdown of the direct and indirect costs associated with PTSD in the UK. The findings highlight the significant economic burden of this condition and the importance of effective interventions and support for individuals with PTSD. In summary, while the study has some limitations, it provides a useful starting point for understanding the economic burden of PTSD in the UK. The authors have taken reasonable steps to address the challenges of using non-UK data and have acknowledged the limitations of their approach. Following addressing t issues raised above, the findings can inform further research and policy discussions aimed at reducing the impact of PTSD on individuals, families, and society as a whole.

VERSION 1 - AUTHOR RESPONSE

Reviewer: 1

Dr. Iain McGowan, QUB

Comments to the Author:

Thank you of asking me to review this paper, and also for accommodating my other commitments to allow extra time for the review. The authors seek to calculate the societal cost of PTSD in the United Kingdom using the 20/21 financial year as a reference year. In order to do so they identify the prevalence of PTSD the UK, as well as identify salient cost categories through a literature search. Extrapolation from the included literature provides an estimate of a societal cost of circa £40000/ per person with PTSD in the UK. This they estimate extrapolates to a total societal burden in excess of £100 billion which they acknowledge is likely to be an underestimate. In general I think the authors have attempted to address a very pressing issue and have made a good attempt at doing so. There are however several issues with the paper that I think should be addressed prior to publication.

I found this paper, at times, a difficult paper to read. I think the paper is too long (circa 10,000 words) and there is a degree of over complexity in the methods used and it can be difficult to follow the authors decisions and processes used in the paper. There are also some inconsistencies in the paper that if addressed should enhance the paper.

On reflection, we agree with this point and have made considerable simplifications of the points made in line with the helpful and detailed comments made below. The paper is now a great deal shorter. We have removed most of the prevalence material agreeing

that this is not needed here. We have made the introduction, results and discussion more structured and easier to read and hope that it now conveys the important findings in a more readable way.

The introductory section attempts to set the scene in terms of the international literature around PTSD and is very detailed and becomes confused, particularly around page 3 para 2 where the authors start to detail how they calculated the UK prevalence of PTSD. I would have expected this in the methods section and not in an introductory section. In this vein, the methods used to calculate the prevalence rate seem very complex. The authors draw the reader's attention to the papers by Scott et al (2022), Foley & Massey (2020) and Yunitiri et al (2021) that report prevalence of PTSD in the UK. Given the three peer reviewed papers in the last four years reporting prevalence of PTSD in the UK it is not clear why the authors chose to compute their own prevalence rate. A formula is provided but it is not clear which figures are used to calculate the final prevalence rate. I am also unclear on the relevance of the international literature reported in the context of detailing what was done to estimate prevalence of PTSD in the UK.

*Thank you for pointing this out. We have moved the calculations to the methods section and report the average prevalence of PTSD. We make clear how this average is calculated. The reason we need to do this is because PTSD significantly differs cross civilian and non-civilian groups and we had to use a weighted average. None of the papers provide this. The papers we mention look only at prevalence across specific groups, Scott et. al look at prevalence for healthcare workers, Foley and Massey look at prevalence for police, and Yunitri et al. examine pooled global estimates of prevalence during COVID-19, an atypical situation which may overstate cost components due to increased alcohol abuse (see Magri et al. 2023) as well as increase in direct costs due to the presence of potential comorbidities. While it is not possible to separate across all categories, we are aware of significant differences in PTSD across the military population and civilian population so have taken a weighted average across that from online websites such as ptsduk and ONS (calculated as civilian population * civilian prevalence rate + military population * military prevalence rate, which stands at approx. 4% for 20/21). We agree that the international material is redundant and have removed it.*

Also, in relation to the introduction I am not sure what relevance the discussions on treatments for PTSD adds to the paper. This is a personal issue and doesn't detract from the overall quality of the paper, but acknowledging my contention that the paper is too long, the authors may want to reconsider how they present this information. Related, the authors correctly assert that there are four medications suggested for treatment by the APA, however to the best of my knowledge only paroxetine and sertraline are

licensed in the UK for treating PTSD. I think it would be appropriate to draw readers attention to that.

Thanks- we agree and have removed much of this section which has helped reduce the length of the paper overall as well as clarifying a great deal. Additionally, there is now increased clarity relating to medications prescribed for PTSD, we did not intend to imply multiple SSRIs would be given at the same time, so appreciate your note.

In the methods section, there are some issues with consistency and I think the purpose of the review of the valuations is lost in the narrative. Page five suggests that the review is to be used to identify categories to decompose the costs associated with PTSD. I assume this means that they will look at what others have costed and use this as a guide to identity cost categories for they analysis. It is not clear, firstly, if this search is conducted as a systematic literature review. If so, I would suggest that the review is incomplete. Arguably at a minimum the PTSD Pubs database should be included in any search strategy-regardless of the presentation or otherwise as a systematic review. I would also suggest that at least one database with a psychological focus (PsychInfo or PsychLit) should be part of the search strategy. I would also question the relevance of EMBASE given the nature of the paper and pharmaceutical focus of EMBASE.

The consistency between the narrative and the systematic review search terms has been recognised and the searches corrected and re-run to check whether papers were missed. This included the mesh terms. You will see the impact of this in Table 1. We added PsycInfo and PTSD Pubs to the search strategy.

Secondly, the search strategy "involved looking for systematic reviews…." (p5). This is not expanded upon in the inclusion criteria section. If it is the case that only systematic reviews are to be included then it would suggest that none of the papers in the review meet the inclusion criteria. Again, on consistency, the narrative on page 5 suggests the terms 'productivity losses' is one of the search terms but this does not appear in the search strategy. Likewise, I would have expected other PTSD associated MESH terms to be included to ensure a fuller representation of the current evidence base.

We have corrected the wording to clarify that we were looking for papers on costs of PTSD including reviews. See points above related to the search.

Thirdly, I understand why the authors have sought to broaden the evidence in relation to cost categories given the relative paucity of evidence in relation to PTSD, however I need more convincing that the health conditions chosen are the most relevant given that PTSD is associated with a myriad of other mental and physical health conditions including schizophrenia and some cancers as well as those highlighted by the authors. It would also be helpful if an additional column could be added to table 1 detailing the cost categories identified from the included studies. It is not clear from the paper the additional benefit of exploring these non-PTSD health conditions over simply looking at

the three included papers focussing on PTSD. An additional column would help in this regard.

This is a very important point and reflected our thinking at that time as we found very few papers on the economic costs of PTSD and were trying to ensure it captured all cost categories so chose a few well analysed health conditions to ascertain what sort of cost components it contained. However, the analysis of these papers mainly were reassuring for us that no important cost category was missed. Hence, the papers are dropped from the Table and we discuss why we did a wider search though it turned out that by collating the categories across the various PTSD papers, we were able to capture the appropriate cost categories. Thus, these papers were more useful for sense checking and we have now revised the paper so it makes this clear and in line with both your comments and that of the second referee. Thus they no longer appear in our search strategy or are included.

The confusion around the aim of the review is compounded in the synthesis section. The justification for looking at other health conditions is that a systematic review of PTSD economic papers (Warth et al, 2020) reported that their included studies were of low to moderate quality. I am not sure that this is a justified criticism given that two of the three PTSD studies the authors include were not reported in the der Warth paper. It is noteworthy that the authors make no comment on the quality of the other health condition papers in their review.

We take this comment on board. The rationale for looking wider is the low number of papers that analyse PTSD and seeing if we can gain insights from the economic burden of other diseases through examining what sort of components are looked at which we have now dropped (see our response to your previous comment). The two papers not included in the review were Bothe et al, 2020; Davies et al. 2022. We have extensively used the categorisation in Davis et. al for non-health care costs. However, as the health care costs were not broken down, we use Ferry et. al for the breakdown. This breakdown was needed as the health care costs for the UK can differ quite a bit from the US so simply converting costs used in Davis would have been inappropriate. Boethe's analysis is for a longer time horizon and so was not directly used.

There are also sections dealing with methodology reported in the synthesis section. Unfortunately, I think this section needs completely rewritten and, ideally, simplified. For me, it would be simpler for the reader (and probably the authors!) to use the cost categories reported in the three PTSD papers found in their review without reference to the other health conditions unless there is a major reason why they have to be included. The authors may want also to consider restructuring the methods section way from the

systematic review format, rather just report what they did without the constraints of a formal SR.

This is a truly helpful suggestion, one which we have taken up and has simplified the synthesis section greatly. Please see updated Table 1.

The section on the calculation of economic costs of PTSD is good-notwithstanding my concerns above-but again overly complex in its presentation. For example, in the data analysis on page 10 I would expect the two paragraphs directly above the sub heading to be in there, not a discussion on previous literature, which sits better in an introductory section. That said, the level of detail in the cost categories is good and the detail provided is what is needed for the reader to follow the decision making of the authors.

Thank you. Again, we have taken up this suggestion by removing the break and thus linking these two paragraphs as suggested.

Some minor points in relation to cost categories; the cost of psychiatric care and psychology, I think, should be categorised as direct healthcare cost. I am not sure my psychiatrist colleagues would take kindly to being categorised as non health care giving, given that GPs and other Doctors have been classified as health care providers and that they are also GMC regulated. I could be persuaded around counselling but as the brunt of the treatments listed are borne by the NHS they should be reflected as directed healthcare cost. I would argue treatment for drug and alcohol use would also fit into this category.

Thank you. Again, we agree and have changed accordingly.

It appears that the authors appear to have assumed that both Paroxetine and Sertraline will be given to all clients with PTSD. This is highly unlikely for two reasons. Firstly, it would be very unusual for two SSRI's to be prescribed concurrently, so clients would get one or other but not both. Secondly, NICE guidelines indicate that medications should not be offered as a first line treatment for PTSD. Economic modelling also showed SSRIs are less cost effective than EMDR, brief individual trauma-focused CBT or self-help with support (NICE 2018). This doesn't make much difference to the overall results but to maintain some form of clinical credibility some comment needs to be made.

This is correct and resulted from some clumsy wording on our part which has been sorted out. Apologies.

I enjoyed reading this paper, but it was hard work! I would strongly encourage the authors to consider simplifying the paper. These suggestions are made with the intention of improving the paper. I trust they will be received as such.

We are most grateful for the detailed and careful comments provided here. They have helped us improve the paper which we believe is now considerably better than it was when submitted. Thank you.

Reviewer: 2

Dr. E Marseille, University of California Berkeley

Comments to the Author:

Minor comments

Throughout, please make it clear when you are referring to costs of patients with PTSD and when you are referring to the excess costs of patients with PTSD, i.e. the costs attributable to PTSD.

Thank you. This has been done throughout.

Bottom 5 / top 6. Define direct health care costs direct non-health care costs and indirect costs.

Done

Page 10, para 2: Word missing after "excess"?

Apologies. This was meant to read 'excess deaths'

Table 1. Why isn't Warth 2020 included?

The paper was referred to but seems to have been missed in Table 1, please see revised Table 1.

Page . 12. I would have thought that psychiatrists, psychologists and councilors administering cognitive behavioral therapy would all be included in direct medical care costs rather than as a non-health care cost. I am more familiar with the healthcare system in the U.S. where therapy of this type would be included as a direct cost of care. Perhaps conventions are different in the UK; otherwise, please move this to the direct health care cost section.

Thank you. We agree and have adjusted the cost headings.

The manuscript would benefit from a thorough proofreading to address minor grammatical and typographical errors.

In line with the suggestions from the other referee we have done a thorough check to catch these errors and reduced the length of the paper as well as improve its readability. Thanks.

Major comments

Page. 6. The rationale for evaluating the economic costs associated with a wide variety of other (non-PTSD) mental health and non-mental health conditions is unclear. The stated rationale is to alert the authors to categories of costs that might be overlooked in the specific PTSD studies. But if they are not quantified in the PTSD studies how does

this help? Providing general “context” for the actual PTSD analysis is an insufficient rationale. Were any papers other than the PTSD papers listed in table one actually used to make the final estimates? Another way of asking this question is, “Why include anything other than PTSD in #2 of the search strategy”?

Bottom of page 8 through line 50 of p 9: Further to my comments above, It is unclear how this review of the economic impact of non-PTSD conditions contributes to the current analysis. I recommend deleting it from what is already a long paper.

In line with both your and referee 1’s point, we have rethought, and they no longer appear. As we explain in our response to referee 1:

This is a very important point and reflected our thinking at that time as we found very few papers on the economic costs of PTSD and were trying to ensure it captured all cost categories so chose a few well analysed health conditions to ascertain what sort of cost components it contained. However, the analysis of these papers mainly were reassuring for us that no important cost category was missed. Hence, the papers are dropped from the Table 1, and we discuss why we did a wider search though it turned out that by collating the categories across the various PTSD papers, we were able to capture the appropriate cost categories. Thus, these papers were more useful for sense checking; we have now revised the paper so it makes this clear and in line with both your comments and that of the second referee they no longer appear in our search strategy or are included.

Page 11. Hospitalization costs. The explanation of these methods is not entirely clear to me. The text refers to the number of hospital visits due to “PTSD-related mental health issues”. However we know that PTSD, apart from mental health disorders, is implicated in a wide range of chronic diseases.

This is a good point of course and handling the co-morbidities is difficult. We recognised this and included it in para 4 of the introduction and brought it up again in the last para of the discussion. We should note however that the excess socio-economic costs associated with PTSD implicitly capture the costs associated with PTSD and may not just be directly because of PTSD but because of the range of chronic conditions which lead to the socio-economic costs we describe.

Page 11. Medication costs and GP costs. Similar to the point made above about hospitalization costs, why restrict medication costs to two SSRIs since again, PTSD is implicated in a wide range of conditions that require medications? Similarly, why restrict the analysis of doctor visits to GP care and not specialist care as well?

We have included the two medications that are recommended for use in the UK for PTSD by NICE. Specialist care is included in the direct costs by way of psychiatrists,

psychologists, counsellors etc. And other specialists can be found in their appropriate headings.

The general point is, why not simply compare the overall direct healthcare costs of people with PTSD to those without PTSD? It would appear that the current methods would systematically underestimate the true excess costs of direct health care for patients with PTSD. Indeed, the £312 annual cost of direct excess healthcare for a PTSD patient in the UK just seems very low. It represents less than a 10% increase over average per capita healthcare spending in the UK in 2022.

We agree that this cost appears low and say so in the main analysis and discussion sections. There are of course several reasons for this which we hope the rewording of these two sections now makes clearer. In particular, note that following moving any of the direct costs as you suggest have now led to an average per person costs of ££779.71 *(see revised Table 1)*

This is lower than calculated in Davis for instance but US direct health care costs are higher than in the UK and costs reported in Ferry are in line with the cost reported here.

We are further reassured by the view of a recently convened expert committee as reported in Hogan et. al (2021) as under:

'The costs of medication, healthcare, and PSS were approximations based on a recent expert panel review convened in England to review the economic evidence on PTSD. The annual costs of being in the PTSD or no PTSD health states were approximated by the panel as £1173 and £110 per person respectively at 2018 prices.'

Page 15. Calculation of premature mortality. According to the explanation given it appears that the median annual salary of £30,000 was applied for the full life expectancy of 75 years. However, the average retirement age is considerably below 75. Please explain or correct this calculation. Presumably these costs are discounted, but discounting is not mentioned. Table 2 Why no entry of premature mortality cost estimates in the total cost column of Table 2?

This is an important point. The cutoff age from which premature mortality is calculated varies in the literature and 75 is a standard value. Yet, as has been rightly pointed out, as the cutoff is above the retirement age of 67 it brings the issue of how to impute an economic value to the productivity of those above retirement age. Measuring it in terms of their contribution to society in terms of unpaid work is one way to handle this. Another is to look at this as in terms of QALYs lost for the individual with the (discounted) sum of QALYs lost. The value of a UK in the UK is approximately £30,000 which would justify our use of that value throughout all age groups. It also roughly coincides with the UK median income which of course raises the point around retirement age, so we have explained this further in the article. These have different advantages and disadvantages

but in the absence of finer data on age specific income and employment rate, this is a reasonable approximation. We further multiply with the fraction of the employed population to give us the most conservative estimate.

Regards discounting, while we did examine estimates with and without discounting (at the standard rate of 3.5%) the analysis in the paper was incorrect due to a calculation error. The error in the formula was unfortunately not noticed and the incorrect figure was added. Once we average the loss due to premature mortality by the population (there was an error both with not doing this and the weights used) the per person costs are much lower and in line with the per person costs of between $132 and $749 (£101 and £572) as reported in Davis et. al.

In view of the earlier error, we feel that we should accompany this response with a full table of our calculations that looks at several scenarios that also addresses the points made below about varying estimates from Davis and other papers e.g. Ahmadi. These differences come from the population under consideration. We would in the interests of full transparency request the full calculations be included in the online appendix allowing for full replicability and consideration by other researchers looking at costs across different population groups. This would also allow for revision of these estimates by other researchers if any of the estimates from which this is calculated change over time.

A significant limitation is the lack of sensitivity analyses to assess the impact of varying key parameters on the cost estimates. Given the uncertainty surrounding some of the input data, it would be informative to present a range of cost estimates based on different assumptions or scenarios. For example, the single biggest contributor to PTSD costs is the economic losses associated with premature mortality. The authors rely on Davies 2022 which in turn seems to cite Miloyan 2016 for its estimate of excess risk of death of 1.2. However, there are studies such as Ahmadi 2011 that provide higher estimates of the relative mortality risk. Since premature mortality is such a large driver of results, this is likely to be a difference that makes a difference. Providing this and other sensitivity analyses using published confidence intervals from the source articles where possible, would provide a more comprehensive understanding of the potential economic burden of PTSD in the UK. The provision of only the base case estimate of £40,000 is insufficient.

As we explain above, our cost calculation was incorrect. The difference in Davis and Ahmadi comes from the fact that Ahmadi considers the military population, Davis looks at both civilian and military and the 0.6 we took was an unweighted average of the two. We examine several scenarios including separate estimates for military and civilian populations. The several tables that we have allows a researcher to look at different scenarios including changes in population distribution, discount rates and different excess death rates across different population groups.

Overall assessment

Despite these limitations, the study makes a valuable contribution to the literature by providing a detailed breakdown of the direct and indirect costs associated with PTSD in the UK. The findings highlight the significant economic burden of this condition and the importance of effective interventions and support for individuals with PTSD. In summary, while the study has some limitations, it provides a useful starting point for understanding the economic burden of PTSD in the UK. The authors have taken reasonable steps to address the challenges of using non-UK data and have acknowledged the limitations of their approach. Following addressing t issues raised above, the findings can inform further research and policy discussions aimed at reducing the impact of PTSD on individuals, families, and society as a whole.

Thank you. We have now gone to some trouble to carefully simplify the paper with a view to clarifying the points made and take on board the helpful points you and the other referee have provided. We are committed to transparency, and to this end have included additional tables of our calculations. The paper is, we believe and think you will agree, considerably more approachable and concise, while still demonstrating the need for increased examination and awareness of the costs, impacts, and burdens of PTSD. This is a diagnosis that lays at the heart of many other problems on an individual and societal level. We hope this paper increases awareness as well as motivating increased resources, research and psychoeducation.

VERSION 2 - REVIEW

**Reviewer**	**2**
**Name**	**Marseille, Elliot**
**Affiliation**	**Health Strategies International**
**Date**	**27-Dec-2024**
**COI**	

I would characterize the revisions I'm requesting as being somewhere between "major" and "minor". There are many strengths to this study and I want to see it published but there are a couple of issues that still need to be addressed. One is the sensitivity analysis question. I describe this in the review itself so I won't repeat my remarks in more detail here, but I simply don't see the sensitivity analyses that you added.

The revised manuscript is much improved and I appreciate the substantial effort that this represents. The comments that follow focus only on areas that still need further review and revision.

Estimation of direct health care costs

The direct healthcare costs were substantially revised upward in the new version. While the original manuscript estimated £312 in annual direct healthcare costs, the revised version shows approximately £780 (with a range of £635 to £924 per patient). This represents a significant increase that better aligns with expected healthcare utilization patterns. The authors provided more justification for their estimates by referencing several sources and the methodology section was revised to better explain how they calculated excess costs by comparing PTSD patients to non-PTSD controls, particularly drawing from Bothe et al.'s work. However, while this component-based approach is an improvement, this elides the main point – Can't we get at the difference in cost between PTSD and non PTSD patients more directly?

The authors mention in their reviewer responses, Hogan 2021. " . . . the annual cost of being in the PTSD or no PTSD health states were 1,173 and 110 per person respectively at 2018 prices". This implies a significantly higher estimate of the excess costs of PTSD than the upwardly revised estimate offered in the revised manuscript, especially when adjusting these 2018 estimates for inflation. The Hogan 2021 estimates in turn are derived from a NICE 2018 expert pane-based assessment plus Mavrenzoul1 2020. Following Hogan 2021, why not us these NICE / Mavrenezouli figures as the base case estimates? I did not look up these studies so maybe they are less than definitive (though I generally find NICE to be thorough and careful), but so is the method of adding up component cost differentials each of which have their own uncertainties. I would like the authors to either use the figures presented in Hogan 2021 i.e. 1,173 for PTSD and 110 for no PTSD or provide a good explanation for why the current approach is likely to be superior.

Excess versus average costs.

The revised manuscript makes more consistent use of terms like "excess costs" particularly when discussing mortality data and economic burden comparisons. However, there could still be more clarity in some sections about whether costs are total or excess costs attributable specifically to PTSD. Please keep this issue in mind as you make the next round of revisions

Sensitivity analyses

The lack of sensitivity analyses was a major reservation I had about the original manuscript. In their revised submission, the authors say, "*We examine several scenarios including separate estimates for military and civilian populations. The several tables that we have allows a researcher to look at different scenarios including changes in population distribution, discount rates and different excess death rates across different population groups".* I see the Appendices; however, I do not see these

tables. Were they included in a separate document apart from the main PDF I accessed for this review?

In Table 2, I see ranges for a number of important inputs. What is the source of those ranges? In one-way sensitivity analyses the procedure is to assign plausible ranges (where possible based on confidence intervals from the source data), to selected important inputs, and then display how the final outcomes of interest (in this case the total cost estimate) varies with the high and low values of each of those inputs. I don't see this in the current manuscript. If the authors are unfamiliar with how to present one way sensitivity analyses, they should review any good cost-effectiveness analysis to see how to display this information in an economical way.

It would strengthen the paper to also run multivariate sensitivity analysis in which each of the selected inputs is varied simultaneously across a distribution (often gamma distributions for cost estimates) and the cumulative uncertainty is calculated and usually graphically displayed following many (e.g. 10,000) trials. I am not imposing multivariate sensitivity analysis as a requirement for publication. It requires dedicated software and some investment of time to learn it. However one-way sensitivity analyses are critical for readers to understand what the effect of uncertainty on major inputs is on the estimate of the final outcome.

VERSION 2 - AUTHOR RESPONSE

Reviewer 2

Comment 1: The direct healthcare costs were substantially revised upward in the new version. While the original manuscript estimated £312 in annual direct healthcare costs, the revised version shows approximately £780 (with a range of £635 to £924 per patient). This represents a significant increase that better aligns with expected healthcare utilization patterns. The authors provided more justification for their estimates by referencing several sources and the methodology section was revised to better explain how they calculated excess costs by comparing PTSD patients to non-PTSD controls, particularly drawing from Bothe et al.'s work. However, while this component-based approach is an improvement, this elides the main point – Can't we get at the difference in cost between PTSD and non PTSD patients more directly?

The authors mention in their reviewer responses, Hogan 2021. " . . . the annual cost of being in the PTSD or no PTSD health states were 1,173 and 110 per person respectively at 2018 prices". This implies a significantly higher estimate of the excess costs of PTSD than the upwardly revised estimate offered in the revised manuscript, especially when adjusting these 2018 estimates for inflation. The Hogan 2021 estimates in turn are derived from a NICE 2018 expert pane-based assessment plus Mavrenzoul1 2020. Following Hogan 2021, why not us these NICE / Mavrenezouli figures as the base case estimates? I did not look up these studies so maybe they are less than definitive (though I generally find NICE to be thorough and careful), but so is the method of adding up component cost differentials each of which have their own uncertainties. I would like the authors to either use the figures presented in Hogan 2021 i.e. 1,173 for PTSD and 110 for no PTSD or provide a good explanation for why the current approach is likely to be superior.

Response:

Thank you, we are glad you agree that the paper is substantially revised and deals with many of your points.
we take on board the point that one can of course simply take the figures in Hogan et. al (2021) based on Mavranzouli et. al (2020) as given and take that as a base case. The costs are based on an expert panel which has consulted the evidence across various reviews and articles on the costs of PTSD and uses costs from PSSRU and is focused on health care utilisation costs as you mention. Of course, we cannot directly compare our paper with Hogan as they do not consider indirect costs which is a big component of costs.
However, we can do as you suggest for the base case i.e. with the direct health care utilisation costs and we felt it would be really worthwhile to do so. Specifically, as Hogan et. al mention, they include costs from the expert panel such as medication, inpatient hospital stays, outpatient visits, general practitioners, and psychological treatment.
Rather than simply take the costs as given, we retained our component-based approach as we feel it provides a more robust way to calculate costs that can be replicated when one component changes to see what the total changes in costs are. While Hogan et. al does not provide much by way of breakdown, we were able to consult Mavranzouli et. al and update some of the frequencies to better reflect current utilisation patterns and we report a central measure of excess direct costs of £1,118.41 (£840.91-£1,353.02) per patient. This is reassuring as it is very close to the £1063 (1173-110) that is reported in Hogan et. al.
At the same time by listing the various components explicitly, we think it provides a more robust way to calculate costs as it can be replicated when one component changes to see what he total change in costs is. We hope this is acceptable and also gives us a direct excess costs in line with the expert panel view on this.

Comment 2: Excess versus average costs.
The revised manuscript makes more consistent use of terms like "excess costs" particularly when discussing mortality data and economic burden comparisons. However, there could still be more clarity in some sections about whether costs are total or excess costs attributable specifically to PTSD. Please keep this issue in mind as you make the next round of revisions

Response: *This has been checked and clarified. Every cost is 'excess cost', instead of 'total costs', as we calculated the costs incurred specifically due to PTSD as is detailed throughout the paper as we discuss each cost component.*

Comment 3: Sensitivity analyses
The lack of sensitivity analyses was a major reservation I had about the original manuscript. In their revised submission, the authors say, "We examine several scenarios including separate estimates for military and civilian populations. The several tables that we have allows a researcher to look at different scenarios including changes in population distribution, discount rates and different excess death rates across different population groups". I see the Appendices; however, I do not see these tables. Were they included in a separate document apart from the main PDF I accessed for this review?
In Table 2, I see ranges for a number of important inputs. What is the source of those ranges? In one-way sensitivity analyses the procedure is to assign plausible ranges (where possible based on confidence intervals from the source data), to selected important inputs, and then display how the final outcomes of interest (in this case the total cost estimate) varies with the high and low values of each of those inputs. I don't see this in the current manuscript. If the authors are unfamiliar with how to present one way sensitivity analyses, they should review any good cost-effectiveness analysis to see how to display this information in an economical way.
It would strengthen the paper to also run multivariate sensitivity analysis in which each of the selected inputs is varied simultaneously across a distribution (often gamma distributions for cost estimates) and the cumulative uncertainty is calculated and usually graphically displayed following many (e.g. 10,000) trials. I am not imposing multivariate sensitivity analysis as a requirement for publication. It requires dedicated software and some investment of time to learn it. However one-way sensitivity analyses are

critical for readers to understand what the effect of uncertainty on major inputs is on the estimate of the final outcome.

Response:

Thank you for pointing this out, while we reported the range of costs in Table 2, we are now clearer how they were derived and have also added a section on one way sensitivity analysis to discuss these in one place for the reader. The sources were reported in table 2 as per journal guidelines with reference numbers in parentheses.
Also, we see why the tables in the Appendix with calculations on premature mortality were not visible in the submitted version as two columns which calculated the costs were cut off at the edges in the clean version showing productivity losses (discounted and undiscounted) for PTSD. We will check the submitted version but have given a summary here as well so you are able to see what we have done. These are done for various population scenarios-only military, only civilians, military and civilian with actual weights based on estimated prevalence.
For sensitivity analysis for the other costs, we did carry out one way sensitivity analysis either based on the CI's used in the source we cite or where point estimates have been reported (because confidence ranges for this component are not reported in the source papers, we have now also computed the cost range if they varied upwards or downwards by 10% (reported as +/-10%). Thank you for noticing that we have not been clear on how these ranges came from.

For example, following consultation with our team of experts and literature, we considered a duration of approximately 6–12-month range[1] for recovery. This is also consistent with a WHO[2] report that shows that up to 40% of people with PTSD recover within a year. Confidence intervals are particularly important in this context, as recovery rates and assistance/support for PTSD vary widely.
In case of premature mortality, we used the figures in Davis et al., for excess mortality rates and we applied weightings based on PTSD prevalence rates within the UK's civilian and military populations. Further details on this methodology can be found in the appendix under Methodology for Computing Costs of Premature Mortality. We have reported ranges throughout all categories which are presented in Table 2, the sources are numbered per BMJ style guidelines.

Thus, our analysis reflects this variability, and the range for the annual cost of PTSD per person was found to vary from £11,372.98-£16,796.76 with a mean annual excess costs of £14,781.37 per person.
